# Characterizing the scent and chemical composition of *Panthera leo* marking fluid using solid-phase microextraction and multidimensional gas chromatography–mass spectrometry-olfactometry

**DOI:** 10.1038/s41598-017-04973-2

**Published:** 2017-07-11

**Authors:** Simone B. Soso, Jacek A. Koziel

**Affiliations:** 1 Iowa State University, Environmental Science Graduate Program, Ames, IA 50011 United States of America; 2 Iowa State University, Department of Agricultural and Biosystems Engineering, Ames, IA 50011 United States of America

## Abstract

Lions (*Panthera leo*) use chemical signaling to indicate health, reproductive status, and territorial ownership. To date, no study has reported on both scent and composition of marking fluid (MF) from *P. leo*. The objectives of this study were to: 1) develop a novel method for simultaneous chemical and scent identification of lion MF in its totality (urine + MF), 2) identify characteristic odorants responsible for the overall scent of MF as perceived by human panelists, and 3) compare the existing library of known odorous compounds characterized as eliciting behaviors in animals in order to understand potential functionality in lion behavior. Solid-phase microextraction and simultaneous chemical-sensory analyses with multidimensional gas-chromatography-mass spectrometry-olfactometry improved separating, isolating, and identifying mixed (MF, urine) compounds versus solvent-based extraction and chemical analyses. 2,5-Dimethylpyrazine, 4-methylphenol, and 3-methylcyclopentanone were isolated and identified as the compounds responsible for the characteristic odor of lion MF. Twenty-eight volatile organic compounds (VOCs) emitted from MF were identified, adding a new list of compounds previously unidentified in lion urine. New chemicals were identified in nine compound groups: ketones, aldehydes, amines, alcohols, aromatics, sulfur-containing compounds, phenyls, phenols, and volatile fatty acids. Twenty-three VOCs are known semiochemicals that are implicated in attraction, reproduction, and alarm-signaling behaviors in other species.

## Introduction

Survival of great cats is contingent on their use of olfaction to identify prey, distinguish amongst conspecifics, indicate reproductive status, and maintain territory among many other roles^1–5^. Unlocking components of excretions that are used as ‘chemical messages’ could lead to reducing human-wildlife conflicts, increasing endangered populations, improving zoological enrichment approaches, and reducing anxiety in captive and wild cat populations. Understanding the roles these chemicals play in behavior could lead to the development of artificial marking sprays using these key semiochemicals that could be used to alter behavior. This has been demonstrated in products for felids such as Feliway®. A recent study by Nace *et al*. (2013) supports the hypothesis that Feliway can lower the fecal corticosteroid metabolites in post-operative artificial insemination procedures for tigers^6^. Researchers have studied scent-marking behaviors and their importance in small cats (*Felis catus*)^7^, pumas (*Puma concolor*)^8^, jackals (*Canis aureus*)^9^, lions (*Panthera leo*)^10^, leopards (*Panthera pardus*)^11–13^, tigers (*Panthera tigris*)^12–14^, and cheetahs (*Acinonyx jubatus*)^13^ to understand the purpose of these markings in animal communication, how they are used for reproduction, territoriality, and enrichment. The marking behavior information gained from these small and great cat studies can be used to increase understanding of how to increase population sizes and prevent great cat extinction. The African lion has experienced devastating decreases in its population over the course of the past 150 years^15^. This indicates a need to restore and prevent the further eradication of the species.

Chemosensory cues play a large role in the reproductive behavior and proliferation of many species. Understanding the role of odors in scent-markings has proven to be integral in the conservation research of a plethora of endangered species. The focus of this work has been to increase reproduction in and out of captivity. Odors within scent-markings have been proven to influence male ejaculation in various animals including giant pandas (*Ailuropoda melanoleuca*)^16^, *Drosophila melanogaster* and *Pieris rapae* ^17–19^, *Zosterisessor ophiocephalus* and *Gobius niger* ^20^, *Gallus gallus* ^21^ and *Microtus pennsylvanicus* ^22^. Males tend to ejaculate in the presence of competitive males in an effort to preserve their genetic influence and survival within their species. In the case of giant pandas it is hypothesized that chemosensory cues from potential rivals “increase male pandas’ sexual motivation towards females, and enhance their territorial behavior”^16^. The lack of competition in captive environments can potentially be inhibiting reproduction of endangered species unless knowledge of chemosensory cues is expanded^16^. Pheromones have been proven to expedite sexual maturity, induce ovulation, reduce post-partum and seasonal anoestrus, and impact copulation in various mammalian species, including rodents, swine, sheep, goats and cattle^23^. Scientific data supports that female animals raised without male contact of the same species will have repressed ovarian function^24^. This has been seen in wildlife species, red deer *Cervus elaphus* ^24^, gray short tailed possum *Monodelphis domestica* ^25^, and mice M*us musculus* ^23^. The pheromone (Z)-7-dodecenyl acetate, found in urine of Asian elephants *Elephas maximus*, has been established as being influential in flehmen behaviors and other pre-mating behaviors^23^. Behaviors of *E. maximus* males toward females and or their urine are strong indicators of oestrous period and receptivity in females, and aggression in males^26, 27^.

Often, studies are able to equate behaviors with scent-markings, and identify specifically the roles of individual compounds in animal behavior in an attempt to understand how the animals are perceiving these scents. The ability of elephants to detect cyclohexanone in musth has led scientists to suspect that some musth signal messages in elephants may be single compounds^28^. More research on the roles of individual scents and chemical compounds within markings is needed to gain an understanding of the influence each has on eliciting behaviors.

Scent-markings are comprised of semiochemicals, which are key components in biota signaling. Lion scent-marks are indicators of their territorial areas, reproductive state, fitness, individuality, genetic variation, and sexual differentiation^13, 24–26^. Lion semiochemicals are excreted through feces, facial rubbing, urine, and marking fluid (MF). However, marking fluid and urine are the most ubiquitous^29–32^. Marking fluid in lions, tigers, leopards, and cheetahs is comprised of urine and a lipid component^24–41^. Lipids are present in the bladder of lions and are released during urination and spray-marking^39^. Andersen and Vulpius^33^ suggested that in *P. leo* these two involuntary methods of marking produce the same range of chemical compounds. The lipid bilayer plays a role in release rate/emissions of volatiles from urine into air^13, 37, 40^. Chemical composition can also be potentially confounded by the direction of release and contact with interfering surfaces^29^. Marking fluid in tigers is known to be sprayed in an upward direction yet in lions it can be varied^32^. If the direction of the released marking changes there is a possibility that urine and marking fluid can be undecipherable.

Although marking behavior in lions has been studied^10^, the chemical and odor composition of lion MF in totality has yet to be investigated. Previously, researchers have chemically characterized volatile constituents of other scent-marking excretions released from lions in their manes^1^, foreheads and cheeks^29^, and urine^33^. Specific compounds are responsible for eliciting behavioral responses, yet studies have generated limited information (i.e., chemical content and scent) on these compounds. This study aims at connecting chemical content of MF with specific scents.

Andersen and Vulpius^33^ suggested that lion urine contained potential traces of MF without additional confirmation^33^. Thus, the presence of 55 VOCs in lion urine was reported^33^. Samples were collected from sawdust bedding in cages^33^. This could have resulted in contamination of samples. To date, no study has reported the composition of total MF (urine + MF) from *P. leo*. The Andersen and Vulpius^33^ study was somewhat limited in the capability of analytical and sample preparation instrumentation because there were compounds reported that were not positively confirmed with chemical standards. The only lion subspecies to have been analyzed for MF VOC composition was *Panthera leo persica* ^32^. However, the main focus of that study was to report on the lack of 2-acetyl-1-pyrroline (2-AP) in anal gland excretion found previously in the MF of Asiatic lions. The focus on 2-AP stems from the earlier finding (Brahmachary, Poddar-Sarkar & Dutta)^40^ that it is a characteristic odor-imparting compound in tiger MF and thought to be in the anal gland fluid of tigers.

This study focused on simultaneous chemical and sensory analyses of total MF, i.e., total as it is released and present in the real environment, without separating into urine and lipid components. The aim was to construct a library of compounds emitted from *P. leo* MF using solid-phase microextraction (SPME) for improved volatiles extraction with minimal matrix interference and multidimensional-GC-MS-olfactometry (MDGC-MS-O) for a comprehensive (both chemical and sensory) analysis. An additional aim was to where feasible, perform standard-based analysis (see Supplementary Information S1). Therefore, the objectives of this study are to 1) develop a novel method for the simultaneous chemical and odor identification of lion MF in its totality, 2) identify the characteristic odorants responsible for the overall scent of lion MF as perceived by human panelists, and 3) compare the results to the existing library of known odorous compounds characterized as eliciting behaviors in animals in order to understand their functionality in lion behavior.

## Results

### SPME Fiber and Time Selection

**Figure 1:**
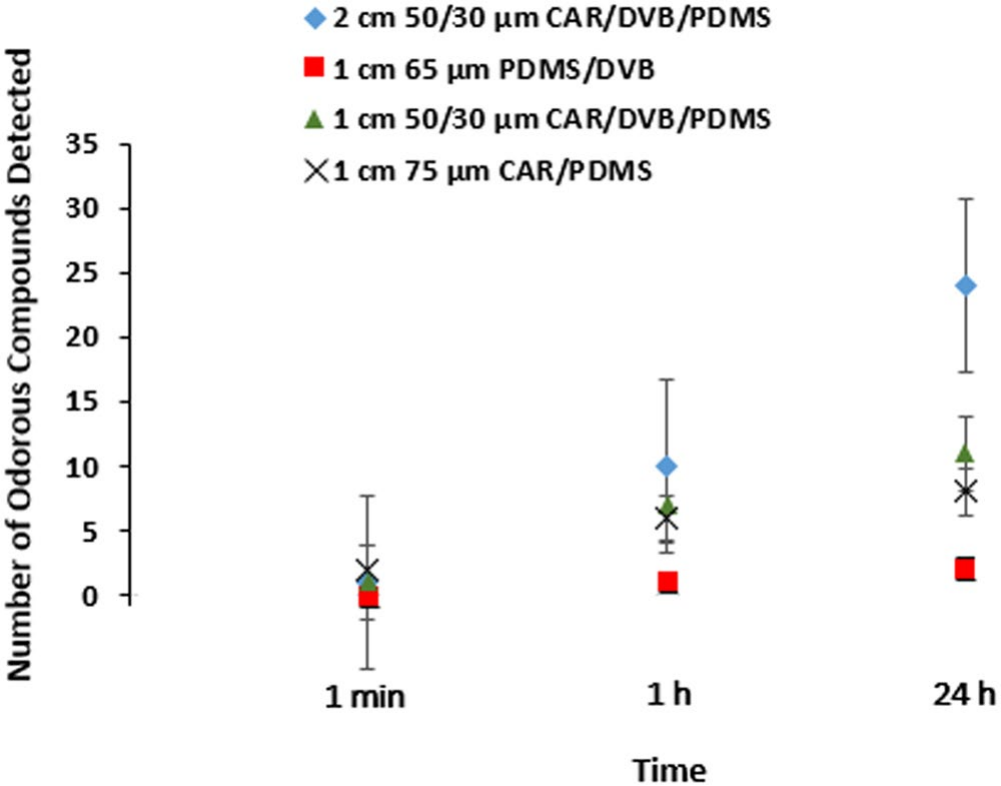
Effects of extraction sampling time (1 min, 1 h, 24 h) and SPME fiber type on the number of odorous compounds detected through sensory analysis (n = 3 replicates) with standard error bars.

Four SPME fiber coatings were compared for volatile organic compound (VOC) extraction efficacy of characteristic MF odorants (see Fig. 1 and Supplementary Table S1). The odor panelists detected 24 odorous compounds with a 24 h sampling time using a 2 cm 50/30 µm Carboxen/divinylbenzene/polydimethylsiloxane (CAR/DVB/PDMS) SPME fiber (see Fig. 1). The average number of compounds detected using a 1 min, 1 h, and 24 h sampling times and all fiber types was 1 ± 0.82, 5.5 ± 3.69, and 11 ± 9.42 respectively. The 2 cm 50/30 µm CAR/DVB/PDMS SPME fiber coating was the most efficient and on average extracted 24 odorous compounds and it was selected for the rest of the experiments. The number of odorous compounds detected increased with sampling time (see Fig. 1). Similarly, an increase of mass extracted by the fiber was observed with increased sampling time (data not shown).

### Identification of Volatile Organic Compounds and Odorants Using MDGC-MS-O

There was a grand total of 81 compounds that were listed as contributing to the total composition of lion MF. A total of 28 VOCs identified in lion MF headspace were confirmed using forward and reverse mass spectral library matches with thresholds of 70% or higher, retention times, and by matching the observed odors detected by panelists against the published odor descriptions for compounds. Out of the 28 confirmed compounds, 7 were organoleptically matched by human panelists and with chemical standard tested confirmed compounds. Twenty-seven of these VOCs were identified with chemical standards (see Table 1). An additional 54 VOCs for which standard confirmation was unavailable have been determined to be volatilized from lion MF (see Supplementary Table S2). Twenty-four of the total 81 compounds were identified through panelist olfactory confirmation and a 24 h SPME extraction (see Table 1, Supplementary Tables S2–3).

**Table 1:** Confirmed VOC odor and chemical composition of *P. leo* marking fluid. All compounds except #28 were confirmed with chemical standards.

No	Compound Name	CAS	Odor Descriptors Observed by Panelists	Measured Odor Intensity	Published Odor Descriptors	Published Odor Detection Threshold (ppb)	Surrogate Odor Activity Value (PA/ODT)	Andersen and Vulpius^33^
1	Trimethylamine^a,†^	75-50-3	Foul, fishy, rancid	100	Fishy, oily, rancid, sweaty^b,c^	3.70–16.00E-01^d^	1.10E + 07	X
2	Acetaldehyde^a,†^	75-07-0	Pungent, chemical, ethereal, and musty	100	Pungent, ethereal, fresh, lifting, penetrating, fruity and musty^b^	1.50-12.00E + 01^d^	1.08E + 04	
3	Acetone^a,ϕ^	67-64-1			Solvent, ethereal, apple, pear^b^	5.00E + 05^d^	5.74E-01	X
4	2-Butanone^a,ϕ^	78-93-3			Ethereal, diffusive and slightly fruity with a camphoreous nuance^b^	5.00E + 04^d^	5.67E + 01	X
5	2-Pentanone^a,ϕ^	107-87-9			Sweet, fruity, ethereal, wine, banana, woody^b^	7.00E + 04^d^	9.12E-01	X
6	3-Hexanone^a,ϕ^	589-38-8			Sweet, fruity, waxy, rum, grape^b^			X
7	Dimethyl disulfide^a,†^	624-92-0	Foul, rotten, vegetable	60	Sulfurous, vegetable, cabbage, onion^b,c^	1.60–120.00E-01^d^	2.14E + 06	
8	3-Methyl-butanal^a,ϕ^	590-86-3			Ethereal, aldehydic, chocolate, peach, fatty^b^	2.50-3.00E + 02^d^	8.40E + 02	X
9	3-Penten-2-one^a,ϕ^	625-33-2			Fruity, acetone, phenolic, fishy^b^	1.53E + 00^d^	1.83E + 05	
10	Heptanal^a,ϕ^	111-71-7			Fresh, aldehydic, fatty, green, herbal, wine-lee ozone^b^	3.00E + 00^d^	1.67E + 04	X
11	Cyclohexanone^a,ϕ^	108-94-1			Minty, acetone^b^	1.20E + 02^e^	2.80E + 03	X
12	Octanal^a,ϕ^	124-13-0			Aldehydic, waxy, citrus, orange peel, green, fatty^b^	7.00E + 01^d^	1.00E + 04	X
**13**	**2,5-Dimethylpyrazine** ^**a**,†^	**123-32-0**	**Nutty, potato, corn, earthy, taco shell, animal, urinous**	**60**	**Musty, potato, cocoa and nutty with a fatty and oily nuance** ^**b**^	**8.00-18.00E + 02 ** ^**f**^	**3.47E + 02**	
14	2-Nonanone^a,ϕ^	821-55-6			Fresh, sweet, green, weedy, earthy, herbal^b^	0.05–2.00E + 02^d^	1.05E + 04	
15	Nonanal^a,ϕ^	124-19-6			Waxy, aldehydic, rose, fresh, orris, orange peel, fatty, peely^b^	2.00E-02^d^	4.66E + 07	X
16	Acetic acid^a,ϕ^	54063-13-7			Sharp, pungent, sour, vinegar^b^	6.00E + 00 ^g^	3.62 + 04	
17	Benzaldehyde^a,ϕ^	100-52-7			Strong, sharp, sweet, bitter, almond, cherry^b^	3.50E + 02-3.50E + 03^d^	3.59E + 03	
18	Linalool^a,†^	78-70-6	Citrus, grassy, green, herbaceous	80	Citrus, orange, floral, terpy, waxy, lavender,rose^b,c^	6.3E + 01^d^	1.60E + 03	
19	1-Octanol^a,ϕ^	111-87-5			Waxy, green, orange, aldehydic, rose, mushroom^b,c^	1.10E + 02-1.30E + 02^d^	3.03E + 03	X
20	Butyrolactone^a,ϕ^	96-48-0			Creamy, oily, fatty, caramel^b^			
21	Acetophenone^a,ϕ^	98-86-2			Sweet, pungent, hawthorn, mimosa, almond, acacia, chemical^b^	6.5E + 01^d^	6.75E + 03	
22	Dodecanal^a,†^	112-54-9	Plastic, waxy	30	Soapy, waxy, aldehydic, citrus, green, floral^b^	2E + 00^d^	7.68E + 04	
23	Phenylethyl alcohol^a,ϕ^	60-12-8			Floral, rose, dried rose, flower, rose water^b^	1.70E + 01 ^h^	1.21E + 04	
24	Phenol^a,ϕ^	108-95-2			Phenolic, plastic, rubber^b^	5.90E + 03^d^	1.27E + 03	X
**25**	**4-Methylphenol** ^**a**,†^	**106-44-5**	**Waxy, herbaceous, butter, sour, animal, barnyard, urinous**	**60**	**Phenolic, narcissus, animal, medicinal, mimosa** ^**b,c**^	**5.50E + 01** ^**d**^	**1.28E + 05**	
26	2-Piperidinone^a,‡,₵^	675-20-7						
27	Indole^a,ϕ^	120-72-9			Animal, floral, moth ball, fecal^b^	1.40E + 04^d^	2.95E + 01	
28	3-Methylcyclopentanone^₵^	6672-30-6	Urinous, sour, animal	30				

*Abbreviations: No-Number; CAS-Chemical Abstract Service Number; PA-Peak Area; ODT-Odor Detection Threshold.

**Compounds in bold are characteristic compounds.

^₵^Compound does not have published odor descriptors, but odor associated with this compounds was detected by panelists.

^ϕ^No odors were detected by panelists, but odor descriptors have been published for this compound.

^‡^No odors were detected by panelists and no odor descriptors have been published for this compound.

^†^Odor descriptors observed by panelists match the published odor descriptors for this compound.

^a^Compounds verified with the retention time and ion confirmation match of standards.

^b^Good Scents Company^98^.

^c^Flavornet^99^.

^d^Leffingwel^100^.

^e^Indoor Air Quality Engineering: Environmental Health and Control of Indoor Pollutants^101^.

^f^Detection thresholds for phenyl ethyl alcohol using serial dilutions in different solvents^102^.

^g^Measurement of Odor Threshold by Triangle Odor Bag Method^103^.

^h^Simultaneous chemical and sensory characterization of VOCs and semi-VOCs emitted from swine manure using SPME and multidimensional gas chromatography-mass spectrometry-olfactometry system^93^.

Odorous VOCs accounted for nearly a third of the total number of VOCs identified and half of the VOCs detected (see Table 1, Supplementary Tables S2–3). An assessor’s breathing cycle can influence detection or sensitivity in olfactometry analysis^42^. Upon exhalation, no odors are being perceived which can cause an odor panelist to miss detection of some compounds^42^. The aqueous and lipid mixture of the MF could be modifying the odor of compounds depending on the distribution of the odorants between the two components^43^. 3-Methylcyclopentanone (tentatively identified with 88% spectral library match) was the only odorous compound organoleptically identified by panelists at the sniff port as having an odor without a published odor descriptor (see Supplementary Table S2, the retention time of 8.59 min). Identifying compounds without previously published odor descriptors allows for potential additions to odor databases. The fact that this compound is without a published odor descriptor does not diminish the impact that it has on the odor of lion MF. There were 20 compounds that had published odor descriptors and were not detected by the panelists (see Table 1, Supplementary Table S3). The inability to detect scents of MF compounds by human panelists further underscores the notion that animals can detect and process a much wider range and even lower concentrations of the same compounds. Cataloging and analyzing scents may provide information for controlled experiments with surrogate scents comprised of odor-active compounds. The present study sought to find out if odor-active compounds are being detected and recognized and their potential roles in lion signaling.

Previously published work on *P. leo* urine suggests that the same compounds are found in both urine and MF. That study^33^ reports 55 compounds of which only 12 were found in this study. One possible reason for this apparent low number of common compounds in both studies is that neither Andersen and Vulpius^33^ nor this present study could confirm the presence of all the compounds detected and they indicated that further confirmation of compounds is necessary. It is important to compare our methods to those used by Andersen and Vulpius^33^ since sample preparation and analysis methods can affect results. Andersen and Vulpius^33^ collected lion urine samples directly from the floor of the night cages. However, due to sawdust contamination, they used a ‘garlic press-like’ device to extract the urine sample, then stored samples in plastic test tubes at −18 °C until analysis. All of these factors, including possible interfering compounds originating from sawdust, may have altered the outcome of earlier findings. An improved characterization of compounds emitted from lion MF without interfering bedding material in this present work, using confirmation with standards and matching of odor descriptors to compounds, has been performed for the first time.

Many of the compounds identified in this study have been identified in the urine and feces of other mammals including African wild dogs (*Lycaon pictus*)^44^, Iberian wolves (*Canis lupus signatus*)^45^, and cheetahs (*Acinonyx jubatus*)^46^. Nearly half of the compounds identified in lion MF were present in African wild dogs (*Lycaon pictus*) urine and feces. Hexanal, octanal, nonanal, acetic acid, benzaldehyde, acetophenone, dodecanal, phenylethyl alcohol, phenol, 2-piperidone, and indole were identified in the MF of lions and the urine of African wild dogs^44^. 2,5-dimethylpyrazine, one of the characteristic compounds of lion MF, was also identified in African wild dog feces^44^. Phenol, 4-methylphenol, and indole were all identified in the MF of lions and the feces of wild Iberian wolves. Martin *et al*. (2010) stated that because indole and phenol are heterocyclic aromatic compounds they aid in increasing the chemical stability of feces from Iberian wolves on surfaces which are humid^45^. This could also be true in the case of lion MF. Due to their ubiquitous presence in most mammals’ urine they are thought to not be species specific^47^. Analysis of cheetah urine led to the confirmation of 27 compounds^46^. Eleven of these 27 compounds (3-methylcyclopentanone, nonanal, phenol, benzaldehyde, octanal, 2-nonanone, dimethyl disulfide, 2-butanone, 3-hexanone, cyclohexanone, 2-pentanone) were also identified in this present study^46^. 3-Methylcyclopentanone, one of the characteristic compounds of lion MF, was confirmed in cheetahs potentially showing a link in great cat scent-marking constituents. This specific compound has not been largely studied in many species. 3-Methylcyclopentanone has also been identified in the urine of badgers (*Meles meles*) and has been used to correlate seasons with reproductive behavior^48^. 3-Methylcyclopentanone has a higher concentration during the autumn mating season in the urine of badgers suggesting that it is related to reproduction in this species^48^.

**Figure 2:**
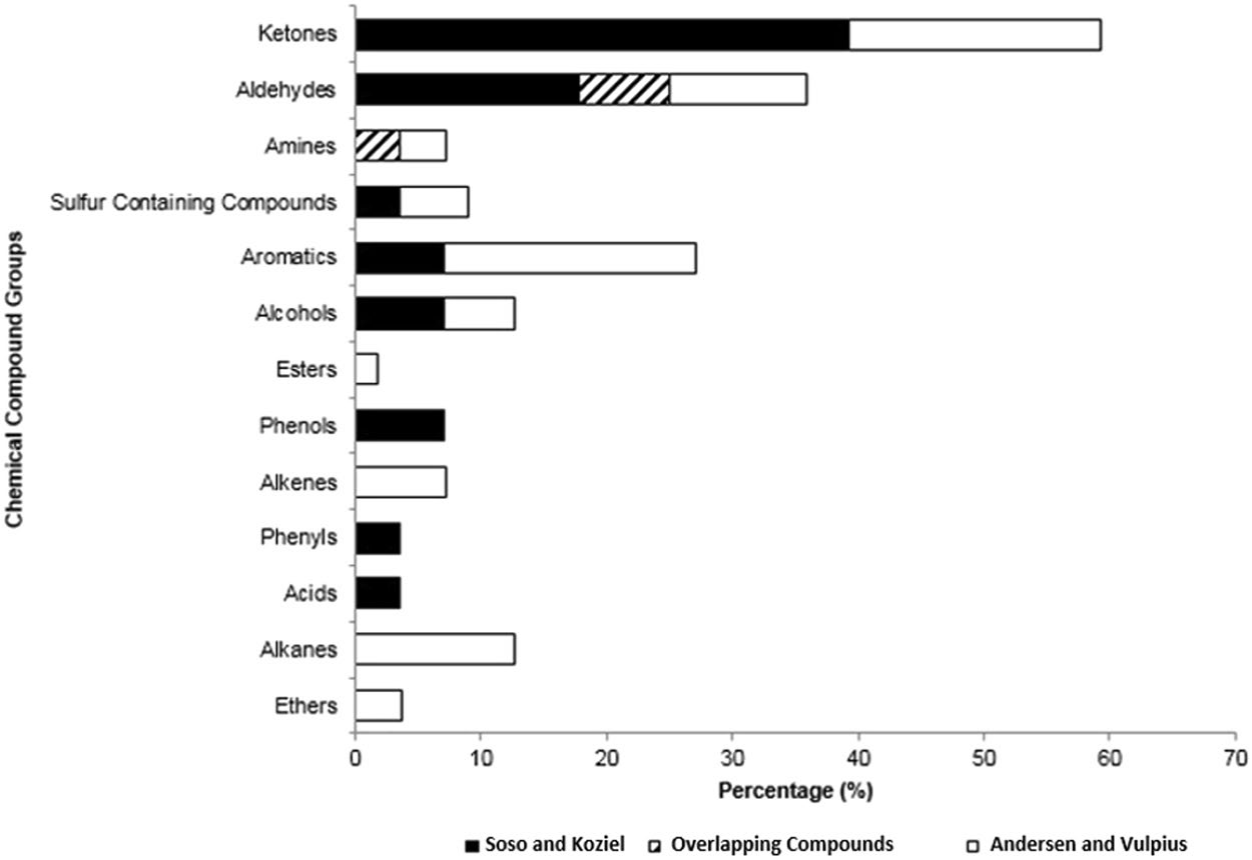
Comparison of marking fluid chemical compound groups. Comparison of the percentage of chemical compound group composition of identified compounds in this study with previously published lion urine compounds (Andersen and Vulpius)^33^.

The use of SPME and MDGC-MS-O made it possible to identify 28 compounds. The following chemical compound groups (and percentages) were present in African lion MF: ketones (39.29%), aldehydes (25%), alcohols (7.14%), aromatics (7.14%), phenols (7.14%), amines (3.57%), sulfur-containing compounds (3.57%), acids (3.57%), and phenyls (3.57%). Figure 2 shows that in comparison with the published literature on *P. leo* urine (Andersen and Vulpius)^33^, three additional chemical compound groups: volatile fatty acids, phenyls, and phenols were identified in this study. Ketones constituted nearly 2x the percentage of the total composition of lion MF in this current study than Andersen and Vulpius^33^ originally identified. Aldehydes and amines contributed equally to the total composition of lion MF in this study and Andersen and Vulpius^33^. Andersen and Vulpius found (7) alkanes, (1) ester, and (2) ethers that were not detected in this study. Also, Andersen and Vulpius^33^ found twice as many alkenes and aromatic compounds compared with this study. One possible explanation is that there was potential contribution of compounds emitted from the sawdust used for cages bedding which was not separated from MF. Compound groups with the highest overlap between this and Andersen and Vulpius^33^ study were aldehydes and amines. Overall, there were 12 compounds identified in MF within this study that were previously unidentified in Andersen and Vulpius^33^.

### Volatile Organic Compounds Responsible for Characteristic Smell of Lion Marking Fluid

The VOCs defined as contributing to the characteristic odor of lion MF were selected based on the relatedness of their odor to the panelists’ perception of the overall aroma of the lion MF. All of the characteristic compounds were detected by panelists and/or had published odor descriptors. Three VOCs define the characteristic odor of lion MF with the characteristic odor descriptors of ‘animal,’ ‘urinous,’ ‘nutty,’ and ‘sour.’ These three characteristic compounds were identified as 2,5-dimethylpyrazine, 4-methylphenol, and 3-methylcyclopentanone (see Table 1, Supplementary Tables S2–3). 3-Methylcyclopentanone was not identified with a standard due to feasibility. 2,5-Dimethylpyrazine and 4-methylphenol were confirmed with chemical standards and spectral matching, while 3-methylcyclopentanone was only tentatively identified using 88% forward and 84% reverse spectral matching (see Table 1, Supplementary Tables S2–3).

**Figure 3:**
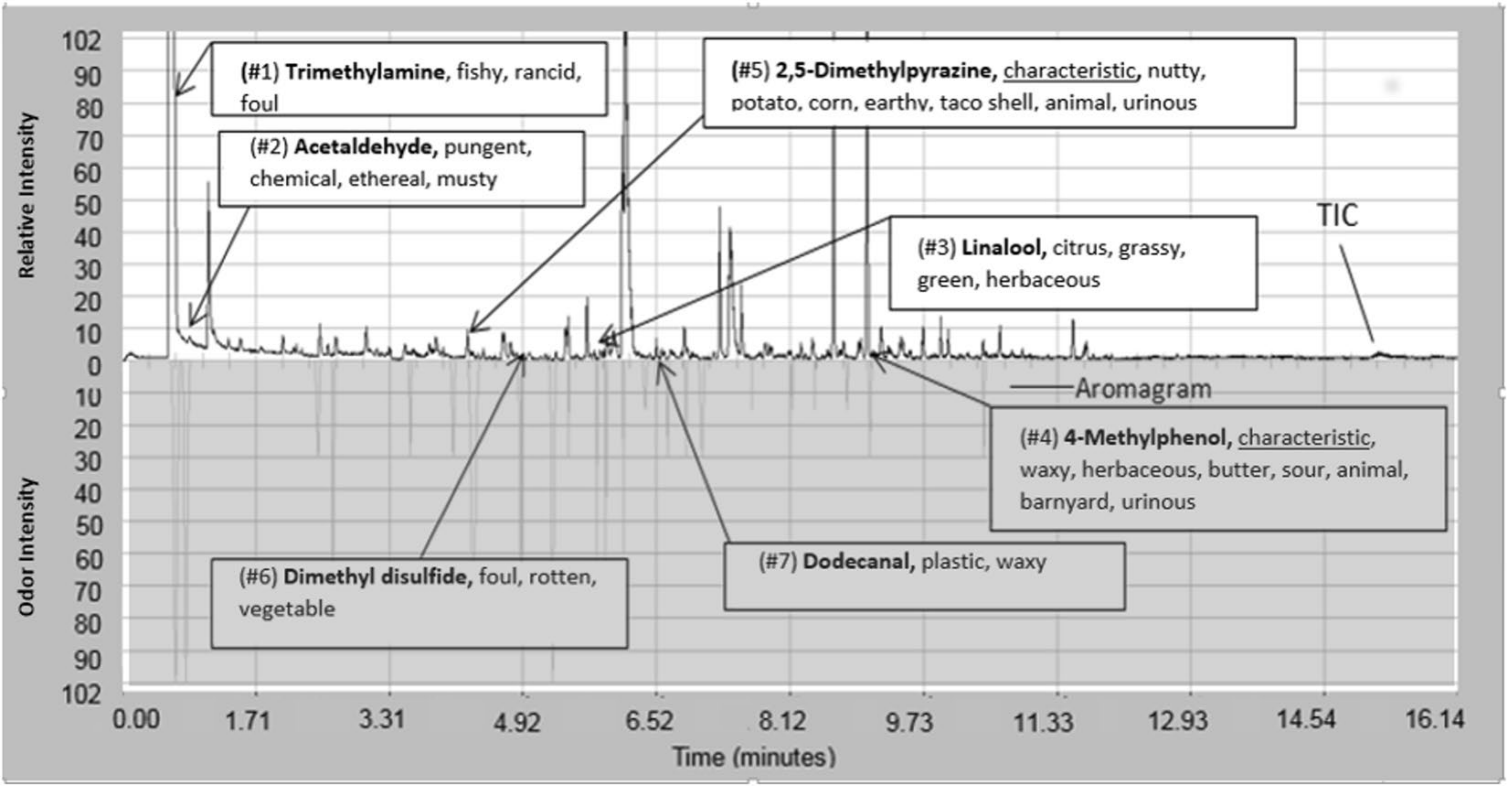
Simultaneous chemical and sensory analyses of compounds and scents in lion MF headspace. Top seven most odorous compounds in lion marking fluid based on measured odor intensity. Chromatogram (top) highlighting identified compounds in lion MF do not necessarily follow their measured odor intensity (aromagram, bottom). The odor character descriptions are based on panelists’ evaluations. Aromagram was created by panelists during sensory analyses, recording odor character, intensity and start-end detection times.

**Figure 4:**
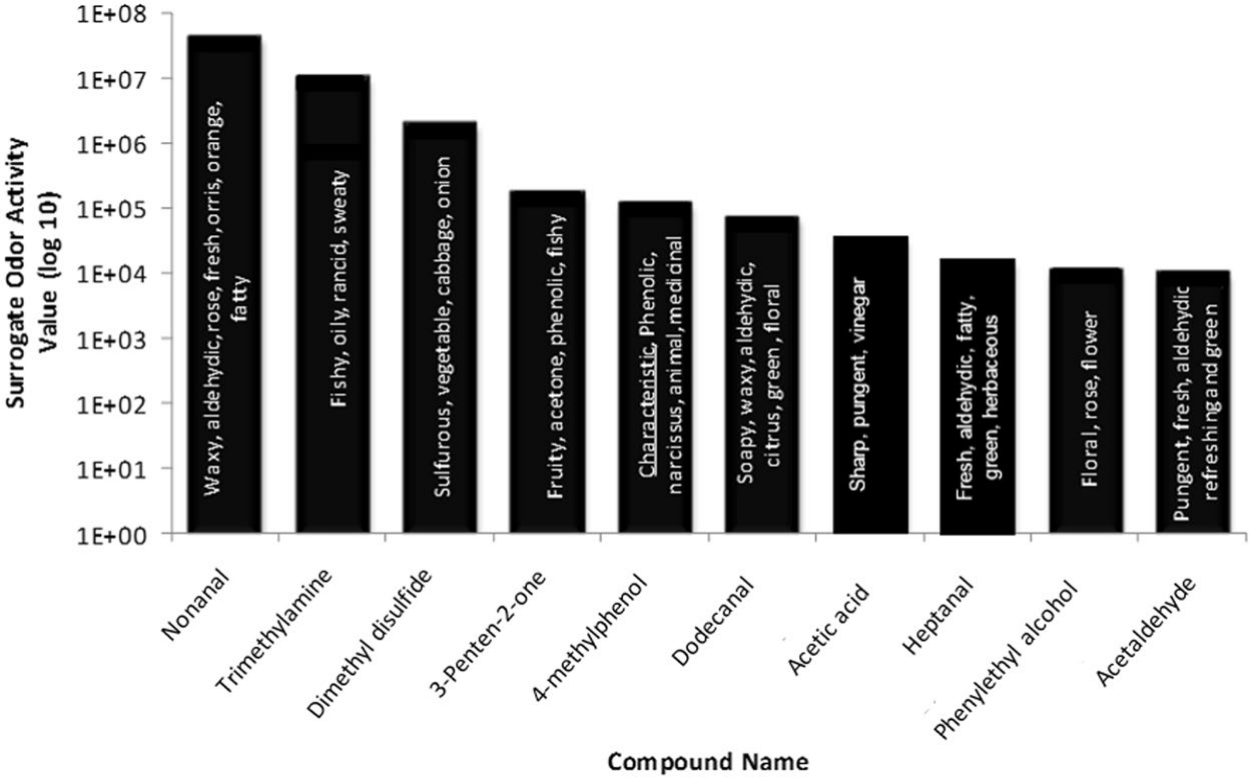
Summary of top 10 identified compounds in lion MF with the highest surrogate odor activity values, OAV (OAV = peak area count/odor detection threshold) and their odor character descriptors.

Surrogate odor activity value (OAV) can be used to describe the impact of an individual compound on the total odor of a sample. It is defined as the ratio of peak area counts and odor detection threshold^49, 50^. The peak area count is used to quantify the amount of the compound’s presence in the total mixture. The odor detection threshold (ODT) is the lowest concentration of a specific odorous compound that is detectable by the human nose. Two of these characteristic odorants (2,5-dimethylpyrazine and 4-methylphenol) have high odor intensities (see example in Fig. 3), yet 2,5-dimethylpyrazine is the only one that has a high surrogate odor activity value (see Fig. 4). Figure 4 ranks the top ten surrogate OAVs limited to those MF compounds for which ODTs are known.

Based on the surrogate OAVs, nonanal, trimethylamine, and dimethyl disulfide are the top three contributing odors to lion MF. The compound with 5^th^ ranked surrogate OAV (4-methylphenol) is one of the characteristic MF odor compounds. Other characteristic compounds were not in the top 10 albeit that does not mean they are lower in odor intensity (see Fig. 3). For example, 3-methylcyclopentanone does not have a published odor detection threshold, thus making it impossible to rank its surrogate OAV. Comparison of the total ion chromatogram (TIC) using heart-cut (HC) mode with the aromagram of lion MF with highlighted peaks with the top seven measured odor intensities within the sample is presented in Fig. 3.

Surrogate OAVs are only able to be calculated if there is a published ODT for a compound. In the absence of an ODT, olfaction can be used to determine the odor intensity of a compound within a sample. This odor intensity can be used to determine the compounds most responsible for the characteristic odor of a mixture. Figure 3 further highlights that high concentration does not necessarily result in a significant odor. Several of the intense scents originate from compounds associated with relatively small TIC peaks (and low relative abundance). The seven odorous compounds in order of rank of odor intensity were: 1) trimethylamine, 2) acetaldehyde, 3) linalool, 4) 4-methylphenol, 5) 2,5-dimethylpyrazine, 6) dimethyl disulfide, and 7) dodecanal. Two (i.e. #4 and #5) of the characteristic odorous compounds were present in the list of highly odor intense compounds demonstrating their importance in imparting the overall odor and could also be affecting mammals’ ability to detect and interpret lion MF. The additional 5 compounds had more of the ‘herbaceous,’ ‘fruity,’ and/or ‘pungent’ odor descriptors. Although speculative at this point, they may be responsible for general detection of lion MF, lion individuality, territoriality, aggression, and indication of desire to copulate.

### The Role of VOCs in Animal Behavior

Twenty-three of the 28 compounds identified in lion MF have been defined as semiochemicals in other animal species (see Table 2). These VOCs play a role in sexual reproduction, receptivity, sex and age differentiation, aggression, attraction, anti-attraction, defense, and locomotion^51–87^. Most semiochemical studies focus on the impact VOCs have on insect behavior. Substantially fewer articles indicate the effect individual VOCs have on large mammal behavior. Studies do indicate that one of the characteristic compounds of lion MF, 2,5-dimethylpyrazine, is also found in wolf (*Canis lupus*) urine and can elicit ‘freezing’ behavior in *Mus musculus* ^67^. This could be indicative of its role in interspecies communication in mammals. The same compound results in aggression in *Locusta migratoria manilensis*, which could be suggestive of a different role in insect communication^68^. 4-Methylphenol is a common component in the urine of many mammals. 4-Methylphenol’s effect on behavior has been thoroughly researched in many animal species *Bubalus bubalis* ^59^; *Alces alces* ^60^; *Glossina* spp.^61–63^; *Stomoxys calcitrans* ^64^; *Equus caballus* ^65, 66^. It plays a primary role in signifying estrus status in *Equus caballus* and *Bubalus bubalis* ^59^ and sexual receptivity in male *Equus caballus* ^65^. 4-Methylphenol is a ubiquitous compound, found in a plethora of animal scent-markings. This compound could be used in behavioral bioassays to understand its role in lion reproduction. Acetic acid is also used as a detector of estrus and copulation signaling in a variety of species^68–72^. Alcohols such as linalool and 1-octanol have been linked to alarm recruitment behavior and attraction, majorly in insects^71, 73–78^.

**Table 2:** VOC composition of *P. leo* marking fluid and published reports of biological role. Bold font signifies compounds responsible for the characteristic odor of lion MF.

No	Compound Name	Cited relevance to behavior	
		Behavior	Species
1	Trimethylamine		
2	Acetaldehyde	Locomotion, Taste aversion, Anxiety	*Rattus rattus* ^52–55^; *Homo sapiens* ^56^; *Mus musculus* ^58–76^
3	Acetone	Locomotion, Sexuality, Irritation	*Rattus rattus* ^104^; *Homo sapiens* ^105^; *Panthera leo* ^33^
4	2-Butanone	Sexuality	*Panthera leo* ^33^
5	2-Pentanone	Reproduction	*Odocoileus virginianus* ^106^
6	3-Hexanone		
7	Dimethyl disulfide	Oviposition inhibition, Attraction, Sniffing	*Anopheles coluzzii* ^84^, *Carollia perspicillata* ^85^, *Rattus rattus* ^86^; *Delia radicum* ^87^; *Glossophaga soricina* ^88^
8	3-Methyl-butanal	Attraction	*Harmonia axyridis* ^107^
9	3-Penten-2-one		
10	Heptanal	Aggregation, Inhibited behavior, Excitation	*Locusta migratoria manilensis* ^68^ *; Culicoides nubeculosus* ^108^ *; Agrotis ipsilon* ^109^
11	Cyclohexanone	Attraction, Locomotion, Stimulation, Inhibition	*Mus musculus* ^110^; *Hyphantria cunea* ^111^; *Steinernema feltiae* ^112^; *Steinernema carpocapsa* ^112^ *; Steinernema kraussei* ^112^; *Heterorhabditis bacteriophora* ^112^
12	Octanal	Immobility	*Mus musculus* ^110^
**13**	**2,5-Dimethylpyrazine**	**Fear, Freezing, Aggression**	***Mus musculus*** ^67^ **;** ***Locusta migratoria manilensis*** ^68^
14	2-Nonanone	Sex attraction	*Leptonycteris curasoae* ^88^; *Rattus norvegicus* ^113^; *Aegorhinus superciliosus* ^114^; *Dendrosoter protuberans* ^115^ *; Cheiropachus quadrum* ^115^; *Ahasverus advena* ^116^
15	Nonanal	Sexual attraction	*Lycaeides argyrognomon* ^81^; gravid *Culex* quinquefasciatus^78^; *Sitotroga cerealella* ^79^ *; Ephestia cautella* ^80^ *; Plodia interpunctella* ^80^ *;Galleria mellonella* ^82^ *; Theraphosa spinipes* ^82^
16	Acetic acid	Estrus, Attraction, Flight	*Bos taurus* ^69, 70^;*Vespula maculifrons* ^71^; *Drosophila melanogaster* ^72^
17	Benzaldehyde	Oviposition, Defensive, Aggression, Alarm recruitment	*Veromessor andre* ^117^; *Scaptotrigona aff. depilis* ^118^; *Nearctic messor* ^119^; *Bombyx mori* ^73^
18	Linalool	Alarm recruitment, Attraction	*Vespula maculifrons* ^71^; *Bombyx mori* ^73^; *Colletes cunicularius* ^74^; *Corythucha cydoniae* ^75^; *Mus musculus* ^76^
19	1-Octanol	Foraging, Alarm recruitment, Sensory perception	*Microplitis croceipes* ^51^ *; Apis dorsata* ^51^
20	Butyrolactone	Appetite, Vomiting, and Tremor Suppression, Estrus	*Papio anubis* ^120^; *Sus scrofa* ^121^; *Bos Taurus* ^121^
21	Acetophenone	Anti-attraction, Attraction, Responsiveness	*Dendroctonus frontalis* ^89^; *Microplitis croceipes* ^122^; *Mus musculus* ^123^; *Dendroctonus brevicomis leConte* ^124^
22	Dodecanal	Physiological Responses	*Culex quinquefasciatus* ^97^
23	Phenylethyl alcohol		
24	Phenol	Estrus, Sexuality	*Idea leuconoe* ^125^; *Bos Taurus* ^70^; *Mamestra brassicae* ^126^; *Bubalus bubalis* ^127^
**25**	**4-Methylphenol**	**Sexuality, Estrus, Diestrus, Sexual attraction**	***Bubalus bubalis*** ^128^; ***Alces alces*** ^60^ **;** ***Glossina spp*** *.* ^61–63^ **;** ***Stomoxys calcitrans*** ^64^ **;** ***Equus Caballus*** ^65, 66^ **;** ***Bison bison*** ^128^
26	2-Piperidinone		
27	Indole	Sexuality, Age differentiation	*Mus musculus* ^129, 130^
**28**	**3-Methylcyclopentanone**	**Seasonal reproduction**	***Meles meles*** ^48^

*Superscripted numbers correspond to the reference source.

Three out of the top five compounds with the highest surrogate odor activity values in lion MF (4-methylphenol, nonanal, and dimethyl disulfide) were also the compounds with most researched olfactory functions and animal behavioral studies^59–66, 78–89^ (see Supplementary Table S3). Their high odor intensity in lion MF could be revealing their importance in lion communication.

### Improved Separation and Isolation of Characteristic Marking Fluid Odorants

**Figure 5:**
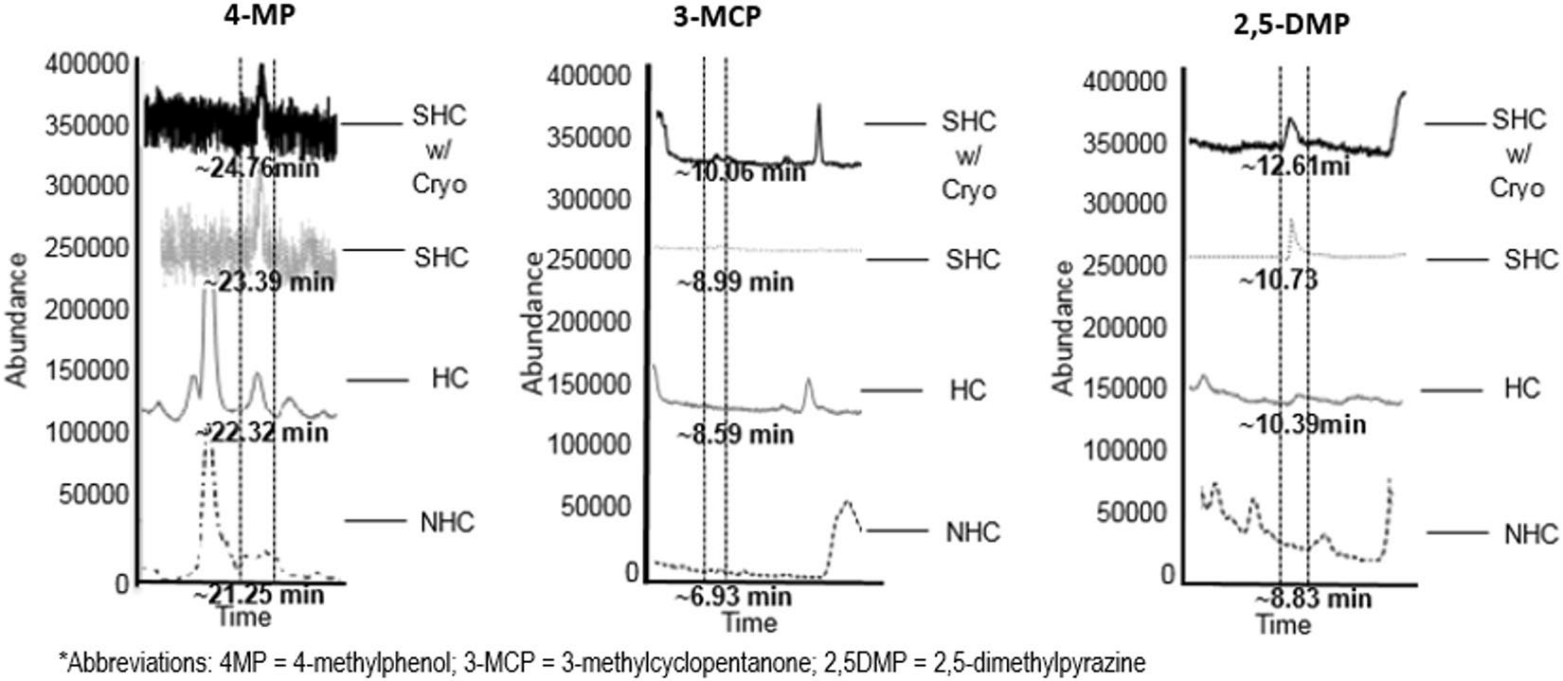
MDGC-MS-O mode for separation and identification of characteristic compounds of lion marking fluid. Separation and enhanced isolation of three characteristic odor-defining compounds extracted from lion marking fluid using four MDGC-MS-O modes: no heart-cut (NHC), heart-cut (HC), Selective Heart-Cut (SHC), and Selective Heart-Cut with Cryotrap (SHC w/Cryo).

Identification of the three key characteristic compounds was performed utilizing 4 different MDGC-MS-O modes: 1) No Heart-Cut (NHC), 2) Full Heart-Cut (HC), 3) Selective Heart-Cut (SHC), and 4) Cryotrap (Cryo) (see Fig. 5). Selective heart-cutting was performed in 30 s increments. The 3 time SHCs occurred at 6.70–7.20 min, 8.60–9.10 min, and 21.00–21.50 min on column 1. These experimental steps were essential to properly isolate the odors and identify areas where the chromatographic peaks may not be apparent (or not separated) but odors are (i.e., they are being detected simultaneously by panelist at the sniff port). Figure 5 shows the improvement in peak resolution as a result of using the four MDGC-MS-O modes. The NHC mode resulted in the aroma identification of 3-methylcyclopentanone (3-MCP) and 4-methylphenol (4-MP). Although the NHC mode produced an odor for 3-methylcyclopentanone and 4-methylphenol, no peak was present in the total ion chromatogram for 3-methylcyclopentanone. Full and selective HC modes were then performed for improved separation and detection of any additional odorous compounds not found in the NHC mode. 2,5-Dimethylpyrazine (2,5-DMP) was identified in addition to 4-methylphenol and 3-methylcyclopentanone in HC mode.

The presence of the aromas at specific retention times indicated where the responsible chromatographic peak should be eluting. The use of n-alkanes aided in determining the ranges in which to perform SHCs for the selected compounds. Selective heart-cutting progressively improved compound identification match with spectral libraries by reducing the background from the sample matrix when the sample was transferred to the analytical (2^nd^) column. The use of HC mode resulted in low percentage matches for 3-MCP (0%), 4-MP (54%), and 2,5-DMP (71%). SHC mode improved the spectral matches, increasing them to 67%, 60%, and 86% for 3-MCP, 4-MP, and 2,5-DMP, respectively. HC-Cryo mode produced the highest percentage matches of all of the four GC-MS modes for 3-MCP, 4-MP, and 2,5-DMP at 84%, 92%, and 97%, respectively. The selective heart-cutting step was necessary to determine if detected odors belonged to more than one coeluting compound. 3-Methylcyclopentanone required the use of cryotrapping to confirm peak identification.

**Figure 6:**
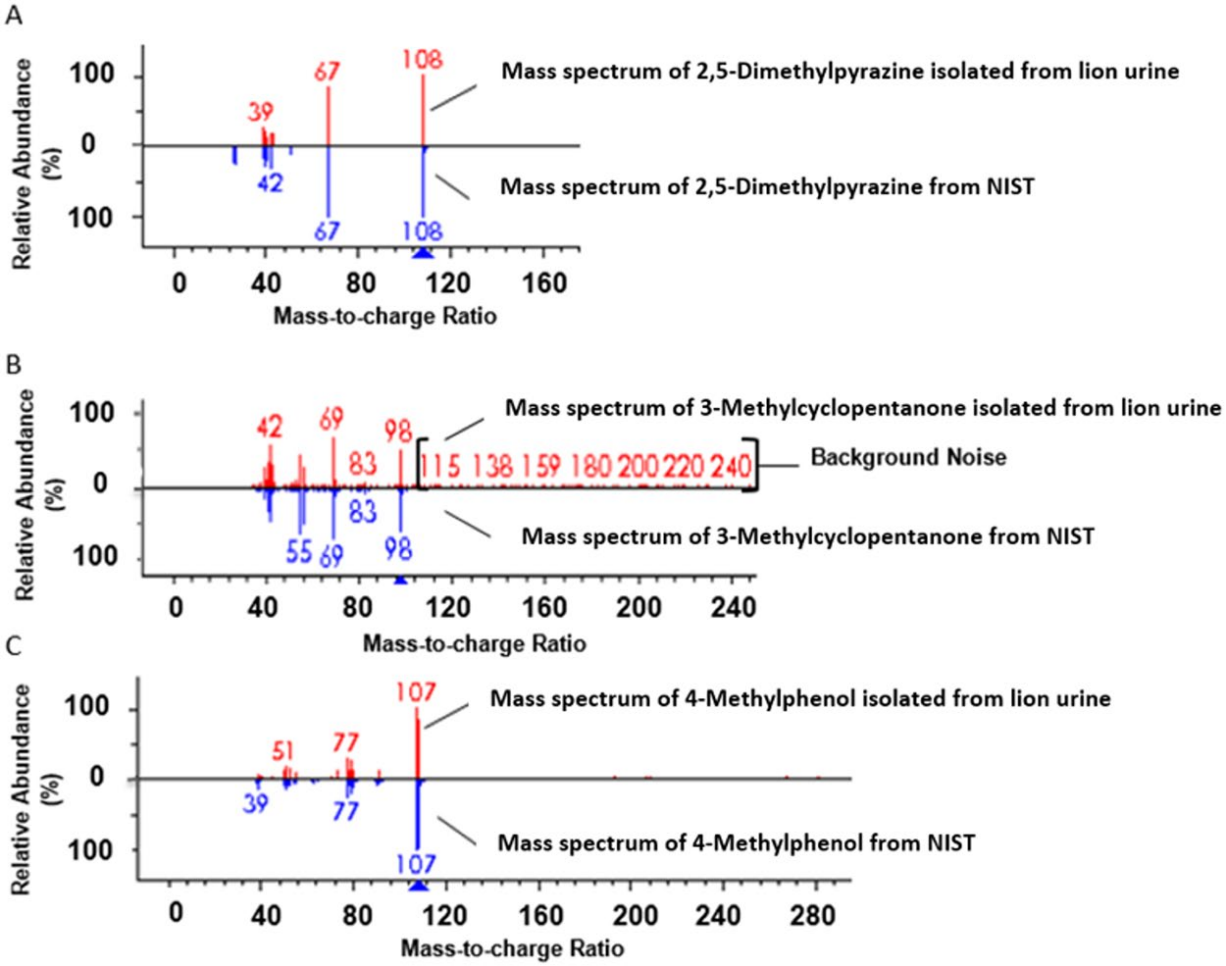
Confirmation of characteristic odorous compounds of lion marking fluid. Mass spectral confirmation of the three compounds responsible for the characteristic odor of lion marking fluid in a Selective Heart-Cut-Cryo mode using the NIST mass spectral library. The relative abundance gives the proportion of ions detected of different masses relative to the largest ion peak.

Cryotrap mode, when activated, was maintained at −40 °C and cooled the short portion of the external front of the analytical column. This cooling process resulted in a peak separation for 3-MP that improved identification with Chemstation, the Automated Mass Spectral Deconvolution and Identification System (AMDIS), and Benchtop Software (see Fig. 6). Without the SHC-Cryo mode, the identification of 3-MP would be less likely. Chemstation, AMDIS, and Benchtop Software programs with a NIST Library found high selected ion and forward and reverse matching for all of the characteristic compounds (see Fig. 6). 3-Methylcyclopentanone was the only characteristic compound that was unable to be confirmed through standard confirmation or published odor descriptors.

## Discussion

This study developed a novel method for the simultaneous chemical and odor identification of lion MF to explore its characteristic odorants. Combining chemical and sensory analysis allowed for the identification of lion MF volatiles that would otherwise be difficult to isolate using typical GC-MS and GC-FID instrumentation. This novel method was able to detect 81 volatile organic compounds, 44 of the 81 compounds were odorous. Three of the 44 odorous compounds were defined as the characteristic compounds in lion MF. The aroma detection of only a third of these confirmed odorous compounds could have been due to their potential low detection thresholds, potential interference from non-volatile components within the sample, the lipid portion of the marking fluid, or from the background contaminants of the enclosure’s floor^43^. The VOCs identified in lion MF play a role in sexual reproduction, sexuality, gender and age differentiation, aggression, attraction, anti-attraction, defense, and locomotion in a variety of species^15, 55, 68, 70, 85, 88, 89^. The 3 compounds with the highest surrogate odor activity values in lion MF (4-methylphenol, nonanal, and dimethyl disulfide) were also the compounds with the most researched olfactory functions in animal behavioral studies. The interest in studying these highly odorous compounds could be due to their pungent smell and ubiquitous nature. Their high odor intensity in lion MF may have a biological role that must be further investigated.

The characteristic odor of lion MF was defined using organoleptics. The characteristic ‘sour,’ ‘urinous,’ ‘animal’ aroma of lion MF is primarily due to three key compounds. The three characteristic odorants are 4-methylphenol, 2,5-dimethylpyrazine, and 3-methylcyclopentanone. Andersen and Vulpius^33^ were unable to detect and identify these three characteristic compounds in urine. This could be indicative of the differences in the analytical advantage of MDGC-MS-O combined with SPME over headspace extraction combined with GC-MS. The use of selective heart-cutting with cryotrap allowed for the probable identification of 3-methylcyclopentanone. Selective heart-cutting created more defined peaks for 3-MCP and 2,5-DMP improving their spectral matches. Future studies should attempt to synthetize a 3-methylcyclopentanone standard to confirm its presence within lion MF. This study did not focus on quantifiably measuring the concentrations of the chemical components of lion MF. Therefore future studies could be performed to determine the exact concentrations of these VOCs. This would aid in understanding at what concentrations the signals are being excreted by lions and would (*i*) elucidate potential sex differences, (*ii*) increase enrichment behaviors^90, 91^, (*iii*) alter reproductive statuses, and (*iv*) further clarify the semiochemicals responsible for individuality.

Future research should focus on performing an animal behavior study to test the effects of these volatile organic compounds on elicitation of specific behaviors. This could be accomplished by measuring changes in hormones (e.g. cortisol) and behavioral responses to the introduction of known behavior-modifying semiochemicals identified in lion MF and other animal species (i.e. 4-methylphenol and acetaldehyde). This could indicate the particular role of each compound in lion behavior modification. The simultaneous chemical and sensory analyses using MDGC-MS-O method can be potentially useful for identification of odorous components in scent marks of other animals. The use of SPME to collect samples in the field and captivity can also be explored. This unique and novel methodology combining SPME and MDCG-MS-O could be used to understand further the way animals perceive scent-markings and potentially prevent the eradication of many large endangered species.

## Methods

### Experimental Site and Animal Subjects

This study was carried out in the Atmospheric Air Quality Laboratory of Iowa State University (ISU) in accordance with the Guide for the Institutional Animal Care and Use Committee. The protocol was approved by Iowa State University’s Institutional Animal Care and Use Committee (IACUC Log # 4–11–7133-A) and by the Blank Park Zoo in Des Moines, Iowa. One male (4 yr. old) and 1 female (6 yr. old) African lion (*P. leo*) from the Blank Park Zoo donated marking fluid samples.

### Marking Fluid Sample Collection

The indoor lion enclosures of the Blank Park Zoo were power-washed with warm water and scrubbed with a floor squeegee for 20 min to reduce sample background contamination. Water used to wash the floor was collected and analyzed to account for additional background contamination and allow for its separation from MF volatiles. Lion behavioral observations were performed by one trained person to time the release of the marking fluid. This was essential to immediately transfer the animal from one enclosure to the adjacent enclosure so that the sample could be collected promptly. At the Blank Park Zoo, keepers identified that these lions released MF in a downward direction. This behavioral marking direction was important in the determination of when to collect marking fluid from the animals. The downward marking was indicative of a marking fluid release, and the white coloration confirmed its identity. Lions were removed from their enclosures, and MF samples were collected immediately from the floor and pipetted into 40 mL glass vials (Supelco, Bellefonte, PA, USA). The MF released from lions appeared yellow with a white lipid film on the top (see Supplementary Fig. S1) and the amount collected ranged from 10 to 20 mL. The vials were washed with a powdered detergent (Alconox, Inc., NY, USA), rinsed with hot water and deionized water for 10 min, then dried at 140 °C overnight prior to use to assure minimum interference with MF. Any polysiloxanes identified were not included in the total composition of the lion MF mass spectral results. These compounds are associated with SPME fibers and capillary GC column bleeds^92^. Any interfering compounds contributed strictly from the water collected from the floor of the enclosure were also not considered to be a component of total lion MF. These water composition compounds were previously unidentified in lion urine. MF samples were collected intermittently between January 1, 2015, through May 15, 2015. On collection days, samples were retrieved during peak lion activity (7 a.m. to 12 p.m.). After collection, the samples were stored in a cooler with ice packs for transportation, and upon returning to the laboratory, samples were further separated into aliquots of 6 mL each and stored in 40 mL vials at −20 °C until analysis.

### Headspace Solid Phase-Microextraction Sampling of Marking Fluid

There was a total of 612 mL of lion marking fluid utilized for this experiment. Thirty-one samples were derived from a female (n = 1) and 31 additional samples were obtained from a male (n = 1). Samples were run in triplicate for each experiment. Sample vials were heated to 39 °C (internal temperature of a lion) and stirred with a Teflon coated stir bar at 1200 rpm for 30 min. Headspace SPME sampling was conducted with a manual fiber holder. After the SPME needle had pierced the septum of the vial, the SPME fiber coating was exposed to the gases emitted from MF into the headspace and the fiber coating continuously adsorbed VOCs.

### Effects of SPME Sampling Time

Four SPME coatings were tested (see Supplementary Tables S1, S4, Fig. 1) using three gas sampling times for extraction and odor characterization efficiency (1 min, 1 h, and 24 h). The selected extraction time was 24 h (see Fig. 1) to maximize the number of odors and compounds identified. The four fibers that were compared were: 2 cm 50/30 µm CAR/DVB/PDMS, 1 cm 65 µm PDMS/DVB, 1 cm 50/30 µm CAR/DVB/PDMS, and 1 cm 75 µm CAR/PDMS. After the VOCs had been extracted, they were then desorbed from the SPME fiber when inserted at 260 °C into the MDGC-MS-O injector^92^. The combination of one-step sample preparation and SPME sampling offset overall processing time for all of the sampling times.

The 1 min sampling time of MF headspace with SPME resulted in no detection of characteristic odors (see Fig. 1). Therefore, when determining an efficient extraction sampling time for SHC and SHC-Cryo, three additional MF headspace sampling times were compared (1 h, 2 h, and 24 h). The 2 h sampling time was used for both SHC and the SHC-Cryo modes because it was the shortest time that reliably resulted in the chemical and odor identification of compounds of interest.

### Olfactory Analysis

Olfactory evaluations were performed through the sniff port. Depending on the MDGC mode, separated compounds eluting from one of the columns were split at a 3:1 ratio (i.e., three parts delivered to a panelist via sniff port, while the remaining one part was sent to the mass spectrometer (MS) for identification. The temperature of the sniff port was set to 240 °C to minimize odorant losses due to condensation in the capillary leading to the sniff port. The tip of the sniff port had a custom nose cone designed at Iowa State University to better fit the panelists. Humidified air was delivered at 5.7 psi to offset the loss of humidity from panelists’ mucous membranes during analyses. The results from the olfactory evaluations were recorded in the form of aromagrams using Aromatrax software (version 6.0, Microanalytics, Round Rock, TX, USA). The aromagram peak was recorded when an odor event was detected by panelists. During the odor event, panelists were responsible for recording (1) the time in which the odor originates and ends, (2) editable odor character descriptors, and (3) odor intensity. The odor intensity was evaluated on a 0–100% scale with 0% indicating no odor and 100% indicating the strongest odor. Only odors that were consistently detected in every one of the three replicates were recorded. The panelists for this study trained extensively on a variety of samples with odorous VOCs. Two trained panelists analyzed the VOCs of lion MF in this study.

### Separation and Isolation of Odorous Compounds with MDGC-MS-O

The MDGC-MS-O has a two GC column system connected in series that can operate in two main modes: no heart-cut (separations on column 1 only, similar to a common GC) and full or selective heart-cut^93^. Heart-cut is defined as a transfer of a selected range of eluting compounds from column 1, the non-polar pre-column, to column 2, the analytical column. Compounds are ‘heart-cut’ from the switch valve (a.k.a. Deans’ switch) and sent for further separations﻿ on column 2 connected in series with column 1.

The cryotrap (i.e., liquid CO_2_ jet delivered to the outside jacket enveloping the front of column 2) can be used to trap selected heart-cut analytes from column 1 to enhance chromatographic separations on column 2.

The following sequence of approaches was used to maximize separation and isolation of odorous VOCs:

1. no heart-cut (NHC),
2. full heart-cut (HC),
3. selective heart-cut (SHC), and
4. selective heart-cut with cryotrapping between columns (SHC-Cryo)

In NHC mode, the sample was separated on column 1 which was 24 m, 0.53 mm, film thickness; 0.50 µm with 5% phenyl methylpolysiloxane stationary phase (SGE BP5) and analyzed by the flame ionization detector (FID) and simultaneously by olfactometry at the sniff port. This allowed initial identification of eluting target odorants for further separation with HC-based modes. During HC mode, the midpoint heart-cut valve was opened for the pre-determined period that could range from seconds (SHC) to the whole GC run (40 min, ‘full’ HC) to allow transfer of compounds from column 1 to 2. The end of column 2 (30 m, 0.53 mm, film thickness, 0.50 µm fused silica capillary column coated with polyethylene glycol, WAX; SGE BP20) was always splitting effluent to the sniff port and MS for simultaneous chemical and sensory analyses. The panelist at the sniff port received separated analytes either from column 1 or column 2 depending on the mode of separation.

The selected HC time was based on the elution time ranges in which odors had been earlier identified by panelists in NHC mode. This allowed for a narrower range of separated compounds from the column 1 to be transferred to column 2 for better isolation, separation, and compound-odor link identification. Standard C_6_-C_20_ alkanes were separated in HC and NHC modes to aid selection of HC ranges, separation, and compound identification. Selecting particular odor-impacting compounds resulted in a reduction of odorless, less important compounds associated with full HC mode. The use of MD-GC-MS-O reduces the sample background and interferences caused by co-eluting compounds, resulting in improved spectral matches^93–96^ and improved identification of key odorants is matrices such as animal waste.

Several 30 to 60 s wide ranges of HC were tested to narrow down the exact retention time in which the compound eluted on column 1 with subsequent separation using HC, SHC, and SHC-Cryo modes. Ultimately, separation and isolation improved for the characteristic compounds with the use of each of the multiple MDGC modes. The ability to resemble the overall lion MF odor was made possible by performing analysis in the SHC-Cryo mode. Separated compounds that were identified as having a scent similar to the ‘characteristic’ (i.e., defined as ‘nutty,’ ‘sour,’ ‘animal,’ and/or ‘urinous’) MF odor descriptors to that of the total MF odor.

Regardless of the heart-cut mode, the same GC and MS program was used. The GC-MS parameters used were: injector, 260 °C; FID, 280 °C; MSD inlet, 240 °C; sniff port, 230 °C; column, 40 °C initial, 3 min hold, 7 °C min^−1^, 240 °C final, 8.43 min hold; carrier gas, GC-grade helium; total run time, 40 min. The GC operated in constant pressure mode where the mid-point pressure was held at 13 psi and the heart-cut sweep pressure was 7 psi. The FID connected to column 1 was maintained at 280 °C with a H_2_ flow rate of 35 mL min^−1^, an air flow rate of 350 mL min^−1^, and the makeup N_2_ flow rate 10 mL min^−1^. The FID acquisition rate was 20 Hz. Mass to charge ratio (m/z) range was set between 32 and 280 amu. Spectra were collected at a high scanning frequency of 7 scans s^−1^ and the electron multiplier voltage was set to 1400 V.

Multitrax (version 7.00, Microanalytics, Round Rock, TX, USA) software was used to control the timing of the HC valves in the MDGC-MS-O in all modes. A select set of criteria were used in the identification of the total list of compounds: 1) top five ion match confirmation, 2) odor descriptor matching (www.goodscentscompany.com and www.flavornet.org) 3) spectral confirmation with standards (Chemstation, Benchtop, and AMDIS_32 Software), 4) column retention time, and 5) NIST Library spectral matches above a threshold of 70%. Chromatographic peaks without the standard confirmation of chemical compounds were not included in the analysis of this study. However, spectral signatures for the non-confirmed compounds were included in the Supplementary Information section (see Supplementary Table S2). The non-confirmed 54 peaks were recorded with their top 5 matching ions, retention times, odor descriptors observed by panelists, and measured odor intensities. Academic Search Premier and Web of Science scientific databases were used to search individual compounds identified in this study that were recognized in animal behavioral studies. The keywords used were: “behavior”, “pheromone”, “animal”, “mammal”, and ‘the name of the compound of interest’.

## Conclusions

The development of a novel method for SPME and simultaneous chemical and sensory analyses with MDGC-MS-O improved separating, isolating, and identifying MF compounds volatilized to air in lion total MF. The ability to use a solvent-free method reduced the potential interference of solvents on the determination of compounds. The multidimensional capacity of the MDGC-MS-O allowed for selective heart-cutting and cryotrapping, where only GC-MS had previously been used in the identification of lion semiochemicals. SHC and cryo-separation techniques provided isolation of specific compounds of interest for improved spectral matching and identification. This method led to the confirmed identification of 28 VOCs of which 8 were identified by odor panelists. Compounds previously unidentified in lion MF were confirmed to be present in the following nin﻿e chemical groups: ketones, aldehydes, alcohols, amines, aromatics, sulfur containing compounds, phenyls, phenols, and volatile fatty acids. Using multidimensional-gas chromatography-mass spectrometry modes of cryotrapping and selective heart-cutting, 2,5-dimethylpyrazine, 4-methylphenol, and 3-methylcyclopentanone were isolated and identified as three of the compounds responsible for the characteristic odor of lion MF. Twenty-three of the 28 compounds identified in lion MF are characterized as eliciting behaviors in other species. These compounds have been shown to influence reproduction, locomotion, freezing behavior, receptivity, sex and age differentiation, aggression, attraction, anti-attraction, and defense in mammals as well as a host of insects. This provides a great introduction to future studies that could focus on the role of chemical compounds in lion behavior. Simultaneous chemical and sensory analysis methods of scent markings can help scientists to understand wildlife behavior and assist in conservation.

## Electronic supplementary material

Supplementary Information
