# Supplementary material for: Characterizing the scent and chemical composition of Panthera leo marking fluid using solid-phase microextraction and multidimensional gas chromatography–mass spectrometry-olfactometry
Source: Sci Rep. 2017 Jul 11;7:5137. doi: 10.1038/s41598-017-04973-2 (PMC5506057; doi:10.1038/s41598-017-04973-2)
Supplement: Supplementary file 1 — Supplementary Information [file 41598_2017_4973_MOESM1_ESM.pdf]

CHARACTERIZING THE SCENT AND CHEMICAL COMPOSITION OF *PANTHERA LEO* MARKING FLUID USING SOLID-PHASE MICROEXTRACTION AND MULTIDIMENSIONAL GAS CHROMATOGRAPHY–MASS SPECTROMETRY-OLFACTOMETRY

Supplementary Note, Figures and Tables

Simone B. Soso and Jacek A. Koziel

Supplementary Note. Additional information on the utility of solid phase-microextraction and multidimensional-gas chromatography-mass spectrometry-olfactometry for research focused on semiochemicals.

Interfacing solid phase-microextraction (SPME) and multidimensional chromatography with olfactometry provides a unique opportunity to address the knowledge gaps in chemical sensory analysis development for lion MF and to identify the chemicals responsible for the characteristic odor of lion MF. The emissions of volatiles from MF (defined here as a simultaneous and mixed excretion of MF and urine) was analyzed in totality, MF was not separated from urine, in order to improve understanding of the perceived odor of gases emitted from lion MF. We did not analyze fecal excretions, a common form of scent-marking, because in lions defecation can be done at random[1]. This is indicating its potentially lower order in the hierarchy of scent-markings. Although the scope of this study was limited to lion's MF, the same approach could be used for other species, excretions, environments, and with behavioral studies. Once odor and odor-causing compounds in territorial markings are known, this knowledge can be exploited to determine the effects of specific compounds on animal behavioral. Future studies can develop behavioral assays and compare changes in chemical composition of scent-markings to the behavioral responses during the introduction of the odorous compounds identified in this study to lions. This unique and novel methodology combining SPME and MDCG-MS-O could be used to further understand the way animals perceive scent-markings, and potentially prevent the eradication of many endangered species.

Chemosensory cues play a large role in the reproductive behavior and proliferation of many species. Understanding the role of odors in scent-markings has proven to be integral in the conservation research of a plethora of endangered species. The focus of this work has been to increase reproduction in and out of captivity. Odors within scent-markings have been proven to influence male ejaculation in various animals including giant pandas (*Ailuropoda melanoleuca*)[2], *Drosophila melanogaster* and *Pieris rapae*[3-5], *Zosterisessor ophiocephalus* and *Gobius niger*[6], *Gallus gallus*[7] and *Microtus pennsylvanicus*[8]. Males tend to ejaculate in the presence of competitive males in an effort to preserve their genetic influence and survival within their species. In the case of giant pandas it is hypothesized that chemosensory cues from potential rivals "increase male pandas' sexual motivation towards females, and enhance their territorial behavior"[2]. The lack of competition in captive environments can potentially be inhibiting reproduction of endangered species unless knowledge of chemosensory cues is expanded[2].

Often, studies are able to equate behaviors with scent-markings, but do not identify specifically the roles of individual compounds in animal behavior nor attempt to understand how the animals are perceiving these scents. The ability of elephants to detect cyclohexanone in musth has led

scientists to suspect that some musth signal messages in elephants may be single compounds[9]. More research on the roles of individual scents and chemical compounds within markings is needed in order to gain an understanding of the influence each has on eliciting behaviors.

Solid phase-microextraction is a solvent-free, one step sampling/sample preparation technique that has been limitedly used in the sample preparation of large mammalian scent-markings[10,11]. Since its conception in the late 1980s, it has proven to be one of the superior sample preparation techniques available for analytical work in the area of fundamental analytical chemistry, environmental analysis, pharmaceutical, food and forensic analyses[10, 12-16]. SPME is a reusable technique that combines sampling and sample preparation and is suitable for laboratory and field environmental work[17]. The SPME process is facilitated on a polymeric coating that has a high affinity for organic compounds. SPME has been used for sampling of volatile compounds in air[18], livestock odor, breath of animals[19, 20], volatiles inside rumen[21], volatiles emitted by decaying animal mortalities[22], and insect-induced plant volatiles among other applications[23]. Enrichment associated with SPME often leads to significantly improved method detection limits and elimination of artifacts from solvents compared with other sampling and preparation methods[24].

Multidimensional-GC-MS-O is one of the most advanced methods for simultaneous chemical and sensory analysis, enabling volatile organic compound separation and isolation of odor-active compounds. Precise and advanced capabilities to detect trace levels of components is due to its multi-column system which allows for a better separation and identification of volatiles[25] many of which are odorous[26-28]. The olfactometry is enabled by a sniff port which gives odor panelists an opportunity to characterize each separated compound as it is being eluted through one of the selected GC columns. This feature allows for the determination and verification of compounds through chemical (GC column retention times, MS spectral matches) and, simultaneous odor matching confirmation using trained odor panelists and published scent-to-compounds link libraries[29]. There is limited working knowledge of how mammals process odor signals[30,31]. Therefore, the human nose is considered ideal in understanding odor perception in animals because the human sense of smell is capable of distinguishing and recognizing a diverse range of characteristics of volatile compounds[32]. Headspace-SPME and MDGC-MS-O was used in the identification of VOCs from *Panthera tigris altaica* MF[33]. This use of SPME in conjunction with MD-GC-MS-O allowed for aroma recognition and chemical confirmation of 2-AP, which was previously considered one of the characteristic odor compounds of *P. tigris tigris* MF, but could not be identified previously using solely chemical analysis with GC-FID and GC-MS[33]. The objectives of this study were to: 1) develop a novel method for the simultaneous chemical and scent identification of lion MF in its totality, 2) identify the characteristic odorants responsible for the overall scent of lion MF as perceived by human panelists, and 3) compare the existing library of known odorous compounds characterized as eliciting behaviors in animals in order to understand their functionality in lion behavior.

1. Schaller, G.B. The Serengeti lion (ed. Schaller, G.B.)1-480 (University of Chicago Press, 1972).

2. Bian, X. *et al*. Exposure to odors of rivals enhances sexual motivation in male giant pandas. *PLOS One*. **8**, e69889; http://dx.doi.org/10.1371/journal.pone.0069889 (2013).

3. Wedell, N. & Cook, P. Butterflies tailor their ejaculate in response to sperm competition risk and intensity. *P. Biol. Sci*, **266**, 1033-1039 (1999).
4. Thomas, M., & Simmons, L. Male-derived cuticular hydrocarbons signal sperm competition intensity and affect ejaculate expenditure in crickets. *P. Biol. Sci.* **276**, 383-388 (2009).
5. Bretman, A., Westmancoat, J. D., Gage M. J. & Chapman T. Males use multiple, redundant cues to detect mating rivals. *Curr. Biol*. 21, 617–622 (2011).
6. Pilastro, A., Scaggiante, M. & Rasotto, M. Individual adjustment of sperm expenditure accords with sperm competition theory. *PNAS*. **99**, 9913-9915 (2002).
7. Pizzari, T., Cornwallis, C.K., Løvlie, H., Jakobsson, S. & Birkhead, T.R. Sophisticated sperm allocation in male fowl. *Nature*. **426**, 70-74 (2003).
8. delBarco-Trillo, J. & Ferkin, M.H. Male mammals respond to a risk of sperm competition conveyed by odours of conspecific males. *Nature*. **431**, 446-449 (2004).
9. Rasmussen, L.E.L., Lee, T.D., Roelofs, W.L., Zhang, A. & Daves, G.D. Insect pheromone in elephants. *Nature*. **379**, 684–684 (1996).
10. Soso, S.B.; Koziel, J.A.; Johnson, A.; Lee, Y.G.; Fairbanks W.S. Analytical methods for chemical and sensory characterization of scent-markings in large wild mammals: a review. *Sensors*. **14**, 4428-4465 (2014).
11. Burger, B. Mammalian semiochemicals in The chemistry of pheromones and other semiochemicals II topics in current chemistry (ed. Schultz S.) 231–278 (Springer, 2005).
12. Pawliszyn J. Solid phase microextraction: theory and practice 2nd ed. (ed. Pawliszyn, J.) 1264 (Wiley, 1997).
13. Sample Preparation for Gas Chromatography. http://dx.doi.org/10.1002/9780470027318.a5508.pub2 (2015).

14. Augusto, F., Leite e Lopes, A. & Zini, C.A. Sampling and sample preparation for analysis of aromas and fragrances. *TrAC*. **22**, 160-169 (2003).

15. Merkle, S., Kleeberg, K.K. & Fritsche, J. Recent developments and applications of solid phase microextraction (SPME) in food and environmental analysis—a review. *Chromatography*. **2**, 293-381 (2015).

16. Brunetti, A. *et al*. Frog volatile compounds: application of in vivo SPME for the characterization of the odorous secretions from two species of Hypsiboas treefrogs. *Chem. Ecol*. **41**, 360–372 (2015).

17. Pawliszyn, J. Handbook of Solid Phase Microextraction. 1st ed (ed. Pawliszyn, J.). 1-50 (Chemical Industry Press, 2009).

18. Koziel, J.A. & Pawliszyn, J. Air sampling and analysis of volatile organic compounds with solid phase microextraction. *J. Air Waste Manage.* **51**, 173-184 (2001).

19. Spinhirne, J.P., Koziel, J.A. & Chirase, N. Sampling and analysis of VOCs in bovine breath using solid-phase microextraction and gas chromatography-mass spectrometry. *J. Chromatogr. A.* **1025**, 63-69 (2004).

20. Cumeras, R., Cheung, W.H.K., Gulland, F., Goley, D. & Davis, C.E. Chemical analysis of whale breath volatiles: A case study for non-invasive field health diagnostics of marine mammals. *Metabolites.* 4, 790-806 (2014).

21. Cai, L., Koziel, J.A., Davis, J., Lo, Y.C. & Xin, H. Characterization of volatile organic compounds and odors by in-vivo sampling of beef cattle rumen gas, by using solid phase microextraction and gas chromatography-mass spectrometry-olfactometry. *Anal. Bioanal. Chem.* **386**, 1791-1802 (2006).

22. Akdeniz, N., *et al*. Air sampling and analysis method for VOCs in field-scale mortality composting operations. *J. Agric. Food Chem.* **57**, 5658-5664 (2009).

23. Cai, L., Koziel, J.A. & O'Neal, M. Studying plant – insect interactions with solid phase microextraction: screening for airborne volatile emissions of soybeans to the soybean aphid, *Aphis glycines matsumura* (*Hemiptera*: Aphididae). *Chromatography*. **2**, 265-76 (2015).

24. Delbarco-Trillo, J., Burkert, B.A., Goodwin, T.E. & Drea, C.M. Night and day: The comparative study of strepsirrhine primates reveals socioecological and phylogenetic patterns in olfactory signals. *J. Evol. Biol.* **24**, 82-98 (2011).

25. Marriott, P.J., Chin, S.T., Maikhunthod, B., Schmarr, H.G. & Bieri S. Multidimensional gas chromatography. *TrAC*. **34**, 1-21 (2012).

26. Lo, Y.C., *et al*. Simultaneous chemical and sensory characterization of VOCs and semi-VOCs emitted from swine manure using SPME and multidimensional gas chromatography-mass spectrometry-olfactometry system. *J. Environ. Qual.* 2008, 37, 521-534.

27. Bulliner, E.A., Koziel, J.A., Cai, L. & Wright, D. Characterization of livestock odors using steel plates, solid-phase microextraction, and multidimensional gas chromatography–mass spectrometry–olfactometry. *J. Air Waste Manage.* **56**, 1391-403 (2006).

28. Rice, S. & Koziel, J.A. The relationship between chemical concentration and odor activity value explains the inconsistency in making a comprehensive surrogate scent training tool representative of illicit drugs. *For. Sci. Intl*. **257**, 257-270 (2015).

29. Flavornet and human odor space. http://flavornet.org/flavornet.html (2015).

30. Albone, E.S. Mammalian Semiochemistry: The investigation of chemical signals between Mammals 1st ed. (ed. Albone, E.S.) (John Wiley & Sons Limited, 1984).

31. Berns, G.S., Brooks, A.M. & Spivak, M. Scent of the familiar: An fMRI study of canine brain responses to familiar and unfamiliar human and dog odors. *Behav. Proc*. **110**, 37-46 (2015).

32. Delahunty, C.M., Eyres, G. & Dufour, J. Review: Gas chromatography-olfactometry. *J. Sep. Sci.* **29**, 2107-2125 (2006).

33. Soso, S.B. & Koziel, J.A. Analysis of odorants in marking fluid of Siberian tiger (*Panthera tigris altaica*) using simultaneous sensory and chemical analysis with headspace solid-phase microextraction and multidimensional gas chromatography-mass spectrometry-olfactometry. *Molecules*. **21**, 834 (2016).

MDGC-MS-O Mode	Extraction Time	Mean # of Odorous Compounds Identified	STD DEV	RSD%
**No Heart-Cut (NHC)**	**1 min**	0	0	0
	**1 h**	5.33	0.47	8.84
	**24 h**	17.3	1.25	7.2
**Heart-Cut (HC)**	**1 min**	1	0	0
	**1 h**	10	0.82	4.41
	**24 h**	24	0.82	3.4

Supplementary Table S1. Effects of extraction sampling time on number of odorous compounds detected. Effect of extraction time (1 min, 1 h, and 24 h) on the number of odorous compounds able to be detected using NHC and HC modes.

No	RT (min)	Top 5 Ions and Their Relative Intensities	Odor Descriptors Observed by Panelists	Measured Odor Intensity
1	2.75	43(99),58(49),41(28),39(24),72(23)		
2	3.18	133(99),73(62),73(32),132(30),59(27)		
3	3.53	57(99),44(33),41(15),58(14),39(11)		
4	3.72	76(99),44(16),32(14),78(7),38(4)		
5	4.13	44(99),56(75),41(60),43(51),57(46)		
6	5.72	30(99),70(5),44(4),41(4),27(3)	Cardboard, medicinal, body odor, rancid, foul	30
7	6.16	43(99),71(67),41(16),114(15),27(11)		
8	6.27	43(99),71(37),41(12),70(8),14(8)	Herbaceous, plastic	80
9	6.57	81(99),80(90),39(22),53(22),42(20)		
10	7.62	43(99),72(81),57(70),41(64),85(29)		
11	7.75	43(99),72(42),41(19),71(15),39(15)		
12	8.03	69(99),55(93),98(68),42(68),56(65)		
13	8.39	81(99),82(26),53(16),138(14),39(7)		
14	8.48	57(99),86(40),71(33),55(26),56(17)		
**15**	**8.59**	**67(99),54(90),82(90),41(70),81(65)**	**Urinous, sour, animal**	**30**
16	9.20	94(99),67(85),66(20),95(6),68(4)		
17	9.84	41(99),54(68),27(59),55(54	Chemical, cardboard, medicinal, wheat	30
18	10.64	128(99),113(50),99(23),85(13),129(8)		
19	10.79	58(99),135(59),91(49),134(40),196(6)		
20	11.80	122(99),121(82),42(74),39(33),67(23)	Herbaceous, dirt, nutty, earthy	80
21	12.21	73(99),83(26),126(16),111(10),127(1)		
22	12.71	97(99),154(21),98(21),45(5),99(7)	Herbaceous, musty, grassy, earthy, dirt	100
23	13.36	58(99),43(73),71(27),59(24),57(14)	Herbaceous	30
24	13.46	43(99),41(99),57(79),55(55),44(54)		
25	14.03	77(99),106(95),105(95),70(76),202(1)		
26	14.19	83(99),55(73),98(34),139(16),140(2)	Citrus, lemon, fruity	80
27	14.24	55(99),83(88),43(87),29(48),98(46)		
28	14.71	95(99),81(43),124(24),79(20),55(15)		
29	14.79	43(99),56(78),41(61),29(57),57(50)		
30	14.99	58(99),41(5),59(4),43(3),42(3)	Herbaceous, cucumber	60
30	15.46			
31	15.61	73(99),58(79),74(5),59(3),60(1)	Foul, burnt	15
32	16.15	43(99),55(99),41(94),56(82),69(73)		
33	16.25	59(99),31(42),41(42),27(18),29(18)	Herbaceous, potato, nutty,	30

Supplementary Table S2 continued

34	17.29	43(99),73(33),55(21),41(20),44(20)	Cardboard, green pepper, herbaceous, plastic	30
35	17.70	55(99),41(97),43(84),69(62),57(58)		
36	18.22	58(99),41(5),43(4),59(4),42(3)		
37	18.86	132(99),133(84),118(21),117(17),130(12)	Medicinal, grassy, herbaceous	15
38	19.28	71(99),43(74),56(55),27(54),89(52)		
39	20.01	57(99),41(69),43(58),55(52),67(42)	Waxy, butter	15
40	20.68	55(99),69(82),57(75),83(71),56(67)	Medicinal, chemical	15
41	21.62	96(99),95(88),39(56),38(14),29(14)	Sweet, cinnamon, phenol, meat	15
42	22.06	55(99),69(79),56(69),57(68),83(66)		
43	22.14	43(99),41(83),55(68),67(48),84(45)		
44	22.74	30(99),99(80),42(78),41(72),43(69)		
45	24.51	192(99),91(24),165(22),119(16),65(15)		
46	24.73	83(99),82(28),153(25),55(19),156(19)		
47	25.75	149(99),177(21),76(14),65(12),150(12)	Citrus, lemon	30
48	26.27	135(99),107(38),164(12),136(10),95(10)		
49	26.64	117(99),90(25),89(11),118(94),116(58)		
50	26.96	105(99),77(65),182(48),51(23),181(80)		
51	28.47	170(99),169(60),141(24),115(15),171(13)		
52	28.93	60(99),44(72),17(70),43(26),16(14)		
53	29.08	95(99),67(76),152(54),96(53),55(47)		
54	29.64	114(99),91(53),65(15)		

Abbreviations: No-Number; RT-Retention Time

**Compounds in bold is the characteristic compound 3-methyl cyclopentanone

Supplementary Table S2. Summary of all unconfirmed peaks in the chromatogram of *P. leo* MF. Compounds were listed by identifying markers: the top five ions, odor descriptors observed by panelist, and retention time.

No	RT (min)	Compound Name	CAS	R. Match (%)	Match (%)	Top 5 Ions and Their Relative Intensities	Odor Descriptors Observed by Panelists	Measured Odor Intensity	Published Odor Descriptors	Published Odor Detection Threshold (ppb)	Surrogate Odor Activity Value (PA/ODT)	Andersen and Vulpius (2009)	Cited relevance to behavior	
													Behavior	Species
1	1.48	Trimethylamine[a,†]	75-50-3	99	99	58(99), 59(68), 30(32), 42(25), 28(10)	Foul, fishy, rancid	100	Fishy, oily, rancid, sweaty[b,c]	3.70-16.00E-01[d]	1.10E+07	X		
2	1.70	Acetaldehyde[a,†]	75-07-0	85	82	44(99),43(57),42(17),41(7),40(2)	Pungent, chemical, ethereal, and musty	100	Pungent, etherial, fresh, lifting, penetrating, fruity and musty[b]	1.50-12.00E+01[d]	1.08E+04		Locomotion, Taste aversion, Anxiety	*Rattus rattus*[1-4], *Homo sapiens*[5], *Mus musculus*[6,7]
3	2.01	Acetone[a,‡]	67-64-1	89	81	43(99),58(64),15(23),42(9),27(6)			Solvent, ethereal, apple, pear[b]	5.00E+05[d]	5.74E-01	X	Locomotion, Sexuality, Irritation	*Rattus rattus*[8], *Homo sapiens*[9], *Panthera leo*[10]
4	2.56	2-Butanone[a,‡]	78-93-3	94	82	43(99),72(25),29(17),27(8),57(8)			Etherial, diffusive and slightly fruity with a camphoreous nuance[b]	5.00E+04[d]	5.67E+01	X	Sexuality	*Panthera leo*[10]
5	3.50	2-Pentanone[a,‡]	107-87-9	81	81	43(99),29(22),27(21),57(19),86(15)			Sweet, fruity, ethereal, wine, banana, woody[b]	7.00E+04[d]	9.12E-01	X	Concentration	*Odocoileus virginianus*[11]
6	4.78	3-Hexanone[a,‡]	589-38-8	90	65	43(99),57(83),71(51),29(51),27(36)			Sweet, fruity, waxy, rum, grape[b]			X		

Supplementary Table S3 Cont'd

7	5.18	Dimethyl disulfide[a,†]	624-92-0	85	95	94(99),79(57),45(48),46(25), 47(19)	Foul, rotten, vegetable	60	Sulfurous, vegetable, cabbage, onion[b,c]	1.60-120.00E-01[d]	2.14E+06		Oviposition inhibition, Attraction, Sniffing	*Anopheles coluzzii*[12], *Carollia perspicillata*[13], *Rattus rattus*[14], *Delia radicum*[15], *Glossophaga soricina*[13]
8	5.36	3-Methyl-butanal[a,ǂ]	590-86-3	87	66	44(99),43(93),41(90),58(81),29(46)			Ethereal, aldehydic, chocolate, peach, fatty[b]	2.50-3.00E+02[d]	8.40E+02	X	Attraction	*Harmonia axyridis*[16]
9	6.36	3-Penten-2-one[a,ǂ]	625-33-2	91	81	69(99),41(96),43(59),39(55),84(27)			Fruity, acetone, phenolic, fishy[b]	1.53E+00[d]	1.83E+05			
**10**	**8.59**	**3-Methyl cyclopenatanone**	**6672-30-6**	**84**	**88**	**67(99),54(90),82(90),41(70),81(65)**	**Urinous, sour, animal**	**30**					**Seasonal reproduction**	***Meles Meles*[17]**
11	8.78	Heptanal[a,ǂ]	111-71-7	85	75	44(99),43(79),70(75),41(60),55(52)			Fresh, aldehydic, fatty, green, herbal, wine-lee ozone[b]	3.00E+00[d]	1.67E+04	X	Aggregation, Inhibited behavior, Excitation	*Locusta migratoria manilensis*[18], *Culicoides nubeculosus*[19], *Agrotis ipsilon*[20]

Supplementary Table S3 Cont'd

12	9.49	Cyclohexanone[a,ǂ]	108-94-1	82	86	55(99),42(75),98(45),41(37),39(36)			Minty, acetone[b]	1.20E+02[e]	2.80E+03	X	Attraction, Locomotion, Stimulation, inhibition	*Mus musculus*[21], *Hyphantria cunea*[22], *Steinernema feltiae*[23], *Steinernema carpocapsa*[23], *Steinernema kraussei*[23], *Heterorhabditis bacteriophora*[23]
13	9.56	Octanal[a,ǂ]	124-13-0	81	77	43(99),29(90),41(90),44(74),57(65)			Aldehydic, waxy, citrus, orange peel, green, fatty[b]	7.00E+01[d]	1.00E+04	X	Immobility	*Mus musculus*[21]
**14**	**10.39**	**2,5-Dimethyl pyrazine[a,†]**	**123-32-0**	**97**	**91**	**108(99),42(99),39(42),40(29),81(16)**	**Nutty, potato, corn, earthy, taco shell, animal, [illegible]**	**60**	**Musty, potato, cocoa and nutty with a fatty and oily nuance[b]**	**8.00-18.00E+02[f]**	**3.47E+02**		**Fear, Freezing, Aggression**	***Mus musculus*[24], Locusta migratoria manilensis[18]**

Supplementary Table S3 Cont'd

15	11.49	2-Nonanone[a,ǂ]	821-55-6	94	91	43(99),58(91),41(29),71(22),57(22)	Fresh, sweet, green, weedy, earthy, herbal[b]	0.05-2.00E+02[d]	1.05E+04		Sex attraction	*Leptonycteris curasoae*[25], *Rattus norvegicus*[26], *Aegorhinus superciliosus*[27], *Dendrosoter protuberans*[28], *Cheiropachus quadrum*[28], *Ahasverus advena*[29]
16	11.58	Nonanal[a,ǂ]	124-19-6	94	94	57(99),41(96),43(82),29(74),44(69)	Waxy, aldehydic, rose, fresh, orris, orange peel, fatty, peely[b]	2.00E-02[d]	4.66E+07	X	Sexual attraction	*Lycaeides argyrognomon*[30], *Gravid culex* quinquefasciatus[31], *Sitotroga cerealella*[32], *Ephestia cautella*[33], *Plodia interpunctella*[33], *Galleria mellonella*[34], *Theraphosa spi nipes*[35]

Supplementary Table S3 Cont'd

17	12.98	Acetic acid[a,ϕ]	54063-13-7	96	81	59(99),43(93),31(92),60(46),29(37)			Sharp, pungent, sour, vinegar[b]	6.00E+00[g]	3.62+04		Oestrus, Estrus, Attraction, Flight	*Bos Taurus*[36,37], *Vespula maculifrons*[38], *Drosophila melanogaster*[39]
18	13.94	Benzaldehyde[a,ϕ]	100-52-7	97	95	77(99),105(92),106(90),51(51),50(32)			Strong, sharp, sweet, bitter, almond, cherry[b]	3.50E+02-3.50E+03[d]	3.59E+03		Oviposition, Defensive, Aggression, Alarm recruitment	*Veromessor andrei*[40]; Scaptotrigona aff. Depilis[41], *Nearctic messor*[42], *Bombyx mori*[43]
19	14.46	Linalool[a,†]	78-70-6	86	70	41(99),43(99),71(90),55(64),93(64)	Citrus, grassy, green, herbaceous	80	Citrus, orange, floral, terpy, waxy, lavender rose[b,c]	6.3E+01[d]	1.60E+03		Alarm recruitment, Attraction	*Vespula maculifrons*[38], *Bombyx mori*[43], *Colletes cunicularius*[44], *Corythucha cydoniae*[45], *Mus musculus*[46]
20	14.52	1-Octanol[a,ϕ]	111-87-5	91	72	43(99),56(97),41(93),55(89),29(63)			Waxy, green, orange, aldehydic, rose, mushroom[1]	1.10E+02-1.30E+02[d]	3.03E+03	X	Foraging, Alarm recruitment, Sensory Perception	*Microplitis croceipes*[47], *Apis dorsata*[48]
21	15.71	Butyrolactone[a,ϕ]	96-48-0	88	82	42(99),28(75),41(58),29(47),27(41)			Creamy, oily, fatty, caramel[b]				Appetite, Vomiting, and Temor Suppression, Estrus	*Papio anubis*[49], *Sus scrofa*[50], *Bos Taurus*[50]

Supplementary Table S3 Cont'd

22	16.01	Acetophenone[a,ɸ]	98-86-2	97	88	105(99),77(86),51(41),130(33),43(21)			Sweet, pungent, hawthorn, mimosa, almond, acacia, chemical[b]	6.5E+01[d]	6.75E+03		Anti-attraction, Attraction, Responsiveness	*Dendroctonus frontalis*[51], *Microplitis croceipes*[52], *Mus musculus*[53], *Dendroctonus brevicomis leConte*[54]
23	16.83	Dodecanal[a,†]	112-54-9	91	90	41(99),57(99),55(82),43(76),82(63)	Plastic, waxy	30	Soapy, waxy, aldehydic, citrus, green, floral[b]	2E+00[d]	7.68E+04		Physiological Responses	*Culex quinquefasciatus*[55]
24	19.94	Phenylethyl alcohol[a,ɸ]	60-12-8	83	67	91(99),92(56),65(22),122(22),39(12)			Floral, rose, dried rose, flower, rose water[b]	1.70E+01[h]	1.21E+04			
25	21.29	Phenol[a,ɸ]	108-95-2	96	95	94(99),66(39),65(27),39(24),40(12)			Phenolic, plastic, rubber[b]	5.90E+03[d]	1.27E+03	X	Estrus, Oestrus, Sexuality	*Idea leuconoe*[56], *Bos Taurus*[37], *Mamestra brassicae*[57], *Bubalus bubalis*[58]
**26**	**22.32**	**4-Methyl phenol**[a,†]	**106-44-5**	**92**	**92**	**107(99),108(82),77(23),27(20),39(19)**	**Waxy, herbaceous, butter, sour, animal, barnyard, urinous**	**60**	**Phenolic, narcissus, animal, medicinal, mimosa**[b,c]	**5.50E+01**[d]	**1.28E+05**		**Sexuality, Estrus, Oestrus, Diestrus, Sexual attraction**	***Bubalus bubalis***[59], ***Alces alces***[60], ***Glossina spp.***[61-63], ***Stomoxys calcitrans***[64], ***Equus Callabus***[65,66], ***Bison bison bison***[67]
27	22.74	2-Piperidinone[a,‡]	675-20-7	85	75	30(99),99(80),42(78),41(72),43(69)								

Supplementary Table S3 Cont'd

28	26.64	Indole[a,ǂ]	120-72-9	95	82	117(99),90(25),89(11),118(94),116(58)		Animal, floral, moth ball, fecal[b]	1.40E+04[d]	2.95E+01		Sexuality, Age differentiation	*Mus musculus*[68,69]

*Abbreviations: No-Number; R. Match-Reverse Match; RT-Retention Time; CAS-Chemical Abstract Service Number

**Compounds in bold are characteristic compounds and are displaying percentage matches from SHC-Cryo mode

[*]Odor descriptors observed by panelists do not match the published odor descriptors for this compound

[₵] Compound does not have published odor descriptors, but odor associated with this compounds was detected by panelists

[ǂ] No odors were detected by panelists, but odor descriptors have been published for this compound

[‡] No odors were detected by panelists and no odor descriptors have been published for this compound

[†] Odor descriptors observed by panelists match the published odor descriptors for this compound

[a] Compounds verified with the retention time and ion confirmation match of standards

[b]Good Scents Company[70]

[c]Flavornet[71]

[d]Leffingwell[72]

[e]Indoor Air Quality Engineering: Environmental Health and Control of Indoor Pollutants[73]

[f] Detection thresholds for phenyl ethyl alcohol using serial dilutions in different solvents[74]

[g] Measurement of Odor Threshold by Triangle Odor Bag Method[75]

Supplementary Table S3. Total VOC composition of *P. leo* marking fluid and its relationship to behavior

References (listed in **Supplementary Table S3**)

1. Orrico, A., *et al*. Efficacy of d-penicillamine, a sequestering acetaldehyde agent, in the prevention of alcohol relapse-like drinking in rats. *Psychopharm*. **228**, 563-575 (2013).
2. Sánchez-Catalán, M., Hipólito, L., Zornoza, T., Polache, A. & Granero, L. Motor stimulant effects of ethanol and acetaldehyde injected into the posterior ventral tegmental area of rats: role of opioid receptors. *Psychopharm*. **204**, 641-653 (2009).
3. Amit, Z., Brown, Z.W., Rockman, G.E., Smith, B. & Amir, S. Acetaldehyde: a positive reinforcer mediating ethanol consumption. *Adv. Exp. Med. Biol*. **126**, 413–423 (1980).
4. Aragon, C.M., Abitbol, M. & Amit, Z. Acetaldehyde may mediate reinforcement and aversion produced by ethanol. An examination using a conditioned taste-aversion paradigm. *Neuropharm*. **25**, 79–83 (1986).
5. Sanchis, C. & Aragón, C.M. What we drink when we drink? The role of the acetaldehyde in the alcohol consumption. *Adicc*. **19**, 5–11 (2007).
6. Escrig, M.A., Pardo, M., Aragon, C.M. & Correa, M. Anxiogenic and stress-inducing effects of peripherally administered acetaldehyde in mice: similarities with the disulfiram-ethanol reaction. *Pharmacol. Biochem. Behav*. **100**, 404-12 (2012).
7. Quertemont, E., Tambour, S., Bernaerts, P., Zimatkin, S.M. & Tirelli. E. Behavioral characterization of acetaldehyde in C57BL/6J mice: locomotor, hypnotic, anxiolytic and amnesic effects. *Psychopharm*. **177**, 84–92 (2004).
8. Lee, D.E., *et al*. The effects of inhaled acetone on place conditioning in adolescent rats. *Pharmacol. Biochem. Behav*. **89**, 101-105 (2008).
9. Arts, J.H.E., Mojet, J., Gemert, L.J. & Feron, V.J. An analysis of human response to the irritancy of acetone vapors. *Crit. Rev. Toxicol.* **32**, 43-66 (2001).

10. Bauer, H., Nowell, K., Funston, P.F., Henschel, P. & Packer, C. *Panthera leo* The IUCN Red List of Threatened Species 2015. I.U.C.N. 1-6; http://dx.doi.org/10.2305/IUCN.UK.2015-4.RLTS.T15951A79929984.en (2015).

11. Miller, K.V., *et al*. Putative chemical signals from white-tailed deer (*Odocoileus virginianus*): social and seasonal effects on urinary volatile excretion in males. *J. Chem. Ecol*. **24**, 673-683 (1998).

12. Suh, E., Choe, D., Saveer, A. & Zwiebel, L.J. Suboptimal larval habitats modulate oviposition of the malaria vector mosquito *Anopheles coluzzii*. *PLOS One*. **11**, e0149800 (2016).

13. Carter, G.G., Ratcliffe, J.M. & Galef, B.G. Flower bats (*Glossophaga soricina*) and fruit bats (*Carollia perspicillata*) rely on spatial cues over shapes and scents when relocating food. *PLOS One*. **5**, e10808 (2010).

14. Seelke, A.M.H. & Blumberg, M.S. Sniffing in infant rats during sleep and wakefulness. *Behav. Neurosci*. **118**, 267-273 (2004).

15. Ferry, A., Le Tron, S,, Dugravot, S. & Cortesero, A. M. Field evaluation of the combined deterrent and attractive effects of dimethyl disulfide on *Delia radicum* and its natural enemies. *Biol. Cntrl*. **49**, 219-226 (2009).

16. Leroy, P. D., *et al*. Honeydew volatile emission acts as a kairomonal message for the Asian lady beetle *Harmonia axyridis* (Coleoptera: Coccinellidae). *Insect Sci*. **19**, 498–506 (2012).

17. Service, K. M., Brereton, R.G. & Harris, S. Analysis of badger urine volatiles using gas chromatography-mass spectrometry and pattern recognition techniques. *Analyst*. **126**, 615-623 (2001).

18. Shi, W.P., Sun, H.L., Edward, N. & Yan, Y.H. Fecal volatile components elicit aggregation in the oriental migratory locust, *Locusta migratoria manilensis* (*Orthoptera:*

Acrididae). *Insect Sci*. **18**, 166-174 (2011).

19. Isberg, E., Bray, D.P., Birgersson, G., Hillbur, Y. & Ignell, R. Identification of cattle derived volatiles that modulate the behavioral response of the biting midge *Culicoides nubeculosus. J. Chem. Ecol*. **42**, 24-32 (2016).
20. Chaffiol, A., *et al*. Plant odour stimuli reshape pheromonal representation in neurons of the antennal lobe macroglomerular complex of a male moth. *Exp. Biol*. **215**, 1670-1680 (2012).
21. De Ceaurriz, J., *et al*. Octanal Concentration-dependent behavioral changes in mice following short-term inhalation exposure to various industrial solvents. *Toxicol. Appl. Pharmacol.* **67**, 383-389 (1983).
22. Tang, R., Zhang, J.P. & Zhang Z.N. Electrophysiological and behavioral responses of male fall webworm moths (*Hyphantria cunea*) to herbivory-induced mulberry (*Morus alba*) Leaf Volatiles. *PLOS One*. **7**, e49256 (2012).
23. Laznik, Ž. & Trdan, S. Attraction behaviors of entomopathogenic nematodes (Steinernematidae and Heterorhabditidae) to synthetic volatiles emitted by insect damaged potato tubers. *J. Chem. Ecol*. **42**: 314-22 (2016).
24. Osada, K., Kurihara, K., Izumi, H. & Kashiwayanagi, M. Pyrazine analogues are active components of wolf urine that induce avoidance and freezing behaviours in mice. *PLOS One*. **24**, e61753 (2013).
25. Munoz-Romo, M., Nielsen, L.T., Nassar, J.M., Kunz, T.H. Chemical composition of the substances from dorsal patches of males of the Curaçaoan long-nosed bat, *Leptonycteris curasoae* (Phyllostomidae: Glossophaginae). *Acta Chiropt*. **14**, 213-224 (2012).
26. Takacs, S., Gries, R., Zhai, H.M. & Gries, G. The sex attractant pheromone of male brown rats: identification and field experiment. *Angewandte Chem. Intl*. **55**, 6062-6066 (2016).

27. Parra, L., *et al*. Volatiles released from Vaccinium corymbosum were attractive to *Aegorhinus superciliosus* (Coleoptera: Curculionidae) in an olfactometric bioassay. *Envir. Entomol*. **38**, 781-789 (2009).

28. Lozano, C., *et al*. Response of parasitoids *Dendrosoter protuberans* and *Cheiropachus quadrum* to attractants of *Phloeotribus scarabaeoides* in an olfactometer. *J. Chem. Ecol.* **26**, 791799 (2000).

29. Wakefield, M.E., Bryning, G.P., Collins, L.E. & Chambers J. Identification of attractive components of carob volatiles for the foreign grain beetle, *Ahasverus advena* (Waltl) (Coleoptera: Cucujidae). *J. Stored Prod. Res*. **41**, 239-253 (2005).

30. Lundgren, L. & Bergström, G. Wing scents and scent-released phases in the courtship behavior of *Lycaeides argyrognomon* (*Lepidoptera*: Lycaenidae). *J. Chem. Ecol*. **1**, 399 (1975).

31. Irish, S.R., Moore, S.J., Bruce, J. & Cameron, M.M. Preliminary evaluation of a nonanal lure for collection of *Gravid culex quinquefasciatus*. *J. Amer. Mosquito Cntrl. Assoc*. **30**, 37-41 (2014).

32. Fouad, H.A., Faroni, L.R.D., Vilela, E.F. & de Lima, E.R. Flight responses of *Sitotroga cerealella* (*Lepidoptera*: Gelechiidae) to corn kernel volatiles in a wind tunnel. *Arthropod Plant Interact*. **7**, 651-658 (2013).

33. Olsson, P.O.C., Anderbrant, O., Lofstedt, C., Borg-Karlson, A.K. & Liblikas, I. Study attractants of nonanal Electrophysiological and behavioral responses to chocolate volatiles in both sexes of the pyralid moths *Ephestia cautella* and *Plodia interpunctella. J. Chem. Ecol*. **31**, 2947-2961 (2005).

34. Dickens, J.C., Eischen, F.A. & Dietz, A. Olfactory perception of the sex attractant pheromone of the greater wax moth, *Galleria mellonella L*. (Lepidoptera: Pyralidae), by

the honey bee, *Apis mellifera L*. (*Hymenoptera*: Apidae). *J. Entomol. Sci*. **21**, 349-354 (1986).

35. Schorkopf, D.L.P., *et al*. Mandibular gland secretions of meliponine worker bees: further evidence for their role in interspecific and intraspecific defense and aggression and against their role in food source signaling. *J. Exp. Biol*. **212**, 1153-1162 (2009).
36. Sankar, R. & Archunan, G. Identification of putative pheromones in bovine (*Bos taurus*) faeces in relation to estrus detection. *Anim. Reprod. Sci*. **103**, 149-153 (2008).
37. Sankar, R., Archunan, G. & Habara, Y. Detection of oestrous-related odour in bovine (*Bos taurus*) saliva: bioassay of identified compounds. *Anim*. **1**, 1321-1327 (2007).
38. Aldrich, J.R., Zhang, Q.H. & Zhang, A.J. Synergistic chemical attraction of the eastern yellowjacket, *Vespula maculifrons* (Hymenoptera: Cespidae). *J. Entomol. Sci*. **39**, 643653 (2004).
39. Becher, P.G., Bengtsson, M., Hansson, B.S. & Witzgall, P. Flying the fly: long-range flight behavior of *Drosophila melanogaster* to attractive odors. *J. Chem. Ecol*. **36**, 599607 (2010).
40. Wyatt, T.D. Pheromones and Animal Behaviour (ed. Wyatt, T.D.) 391 (Cambridge University Press, 2003).
41. Townsend. G.F. Benzaldehyde: a new repellent for driving bees. *Bee World*. **44**, 146-149 (1963).
42. Holldobler, B. et al. Pygidial gland chemistry and potential alarm-recruitment function in column foraging, but not solitary, *Nearctic messor* harvesting ants (Hymenoptera: Formicidae: Myrmicinae). *J. Insect Physiol*. **59**, 863-869 (2013).
43. Anderson, A.R., *et al*. Molecular basis of female-specific odorant responses in *Bombyx mori*. *Insect Biochem. Mol. Biol*. **39**, 189-197 (2009).

44. Borg-Karlson, A.K., *et al*. (S)-(+)-linalool, a mate attractant pheromone component in the bee *Colletes cunicularius*. *J. Chem. Ecol.* 29, 1-14 (2003).

45. Aldrich, J.R., Neal Jr, J.W., Oliver, J.E. & Lusby, W.R. Chemistry *vis-à-vis* maternalism in lace bugs (Heteroptera: Tingidae): Alarm pheromones and exudate defense in *Corythucha* and *Gargaphia* species. *J. Chem. Ecol*. **17**, 2307-2322 (1991).

46. Cavaggioni, A., Mucignat-Caretta, C. & Redaelli, L. Mice recognize recent urine scent marks by the molecular composition. *Chem. Senses*. **33**, 655-663 (2008).

47. Meiners, T., Wackers, F. & Lewis, W.J. The effect of molecular structure on olfactory discrimination by the parasitoid *Microplitis croceipes*. *Chem. Senses*. 27, 811-816 (2002).

48. Li, J.J., Wang, Z.W., Tan, K., Qu, Y.F. & Nieh, J.C. Effects of natural and synthetic alarm pheromone and individual pheromone components on foraging behavior of the giant Asian honey bee, *Apis dorsata. Exp. Biol*. **217**, 3512-3518 (2014).

49. Goodwin, A. K., *et al.* Behavioral effects and pharmacokinetics of gamma-hydroxybutyrate (GHB) precursors gamma-butyrolactone (GBL) and 1,4-butanediol (1,4-BD) in baboons. *Psychopharmacol*. **204**, 465–476 (2009).

50. Kubelka, M. *et al*. Activation of pig and cattle oocytes by butyrolactone I: morphological and biochemical study. *Zygote*. **10**, 47-57 (2002).

51. Shepherd, W.P. & Sullivan, B.T. Southern Pine Beetle, *Dendroctonus frontalis*, Antennal and behavioral responses to nonhost leaf and bark volatiles. *J. Chem. Ecol*. **39**, 481493 (2013).

52. Li, Y.S., Dickens, J.C. & Steiner, W.W.M. Antennal olfactory responsiveness of *Microplitis-croceipes* (hymenoptera, braconidae) to cotton plant volatiles. *J. Chem. Ecol*. **18**, 1761-1773 (1992).

53. Fleischmann, A., *et al*. Mice with a "Monoclonal Nose": Perturbations in an olfactory map impair odor discrimination. *Neuron*. **60**, 1068-1081 (2008).

54. Erbilgin, N., *et al*. Acetophenone as an anti-attractant for the western pine beetle, *Dendroctonus brevicomis LeConte* (Coleoptera: Scolytidae). *J. Chem. Ecol*. **33**, 817-823 (2007).

55. Cooperband, M.F., McElfresh, J.S., Millar, J.G. & Cardé, R.T. Attraction of female *Culex quinquefasciatus Say* (Diptera: Culicidae) to odors from chicken feces. *J. Insect Physiol*. **54**, 1184-1192 (2008).

56. Nishida, R., *et al*. Male sex pheromone of a giant danaine butterfly, *Idea leuconoe*. *J. Chem. Ecol*. **22**, 949-972.

57. Jacquin, E., Nagnan, P. & Frerot, B. Identification of hairpencil secretion from male *Mamestra brassicae* (L) (Leppidoptera: Nostuidae) and electroantennogram studies. *J. Chem. Ecol.* **17**, 239-246 (1991).

58. Rajanarayanan, S. & Archunan G. Identification of urinary sex pheromones in female buffaloes and their influence on bull reproductive behavior. *Res. Vet. Sci*. **91**, 301-305 (2011).

59. Karthikeyan, K., *et al*. Faecal chemical cues in water buffalo that facilitate estrus detection. *Anim. Reprod. Sci*. **138**, 163-167 (2013).

60. Whittle, C.L., Bowyer, R.T., Clausen, T.P. & Duffy, L.K. Putative pheromones in urine of rutting male moose (*Alces alces*): evolution of honest advertisement? *J. Chem. Ecol*. **26**, 2747-2763 (2000).

61. Hall, D.R., Beevor, P.S., Cork, A., & Vale, G.A. A potent olfactory stimulant and attractant for tsetse isolated from cattle odours. *Insect Sci. App*. 5, 335–339 (1984).

62. Owaga, M.L.A., Hassanali, A. & McDowell, P.G. The role of 4-cresol and 3-normalpropylphenol in the attraction of tsetse flies to buffalo urine. *Intl. J. Trop. Insect. Sci*. **9**, 95-100 (1988).

63. Harraca, V., Sved, Z. & Guerin, P.M. Olfactory and behavioural responses of tsetse flies, *Glossina* spp., to rumen metabolites. *J. Comp. Physiol. A Neuroethol. Sens. Neural. Behav. Physiol*. **195**, 815-824 (2009).

64. Jeanbourquin, P. & Guerin, P.M. Sensory and behavioural responses of the stable fly *Stomoxys calcitrans* to rumen volatiles. *Med. Vet. Entomol*. **21**, 217-224 (2007).

65. Buda, V., Mozuraitis, R., Kutra, J. & Borg-Karlson, A.K. *p*-Cresol: a sex pheromone component identified from the estrous urine of mares. *J. Chem. Ecol*. **38**, 811–813 (2012).

66. Mozuraitis, R., Buda, V., Kutra, J., & Borg-Karlson, A.K. *p*- and *m*-Cresols emitted from estrous urine are reliable volatile chemical markers of ovulation in mares. *Anim. Reprod. Sci*. **130**, 51-6 (2012).

67. Karthikeyan, K., *et al*. Identification of *p*-cresol as an estrus-specific volatile in buffalo saliva: comparative docking analysis of buffalo OBP and *β*-Lactoglobulin with *p*-cresol. *Zool. Sci*. **31**, 31-36. 121 (2014).

68. Osada, K., *et al*. The scent of age. *Proc. R. Soc. Lond. B*. **270**, 929–33 (2013).

69. Nicole, J., Gläser, F. & Bräse, S. Synthetic approaches to polycyclic semiochemicals and their derivatives: combinatorial methods towards phytochemicals. *Phytochem. Rev*. **12**, 603–651 (2013).

70. Good Scents Company. *Good Scents Company Information System* http://www.thegoodscentscompany.com/ (1994).

71. Acree, T. & Heinrich, A. *Flavornet and human odor space*. http://flavornet.org/flavornet.html (2015).

72. Leffingwell & Associates. *Odor Detection Thresholds* http://www.leffingwell.com/odorthre.htm (2012).

73. Heinsohn, R. & Cimbala J. Indoor air quality engineering: Environmental health and control of indoor pollutants 1st ed. (Upper Saddle River, 1999).

74. Tsukatani, T., Miwa, T., Furukawa, M. & Costanzo, R. Detection thresholds for phenyl ethyl alcohol using serial dilutions in different solvents. *Chem. Senses*. **28,** 25-32 (2003).

75. Nagata, Y. Measurement of odor threshold by triangle odor bag method in Asian network on odor measurement and control (ed. Nagata, Y.) 118-127 (Tokyo Japan Association On the Environment, 2013).

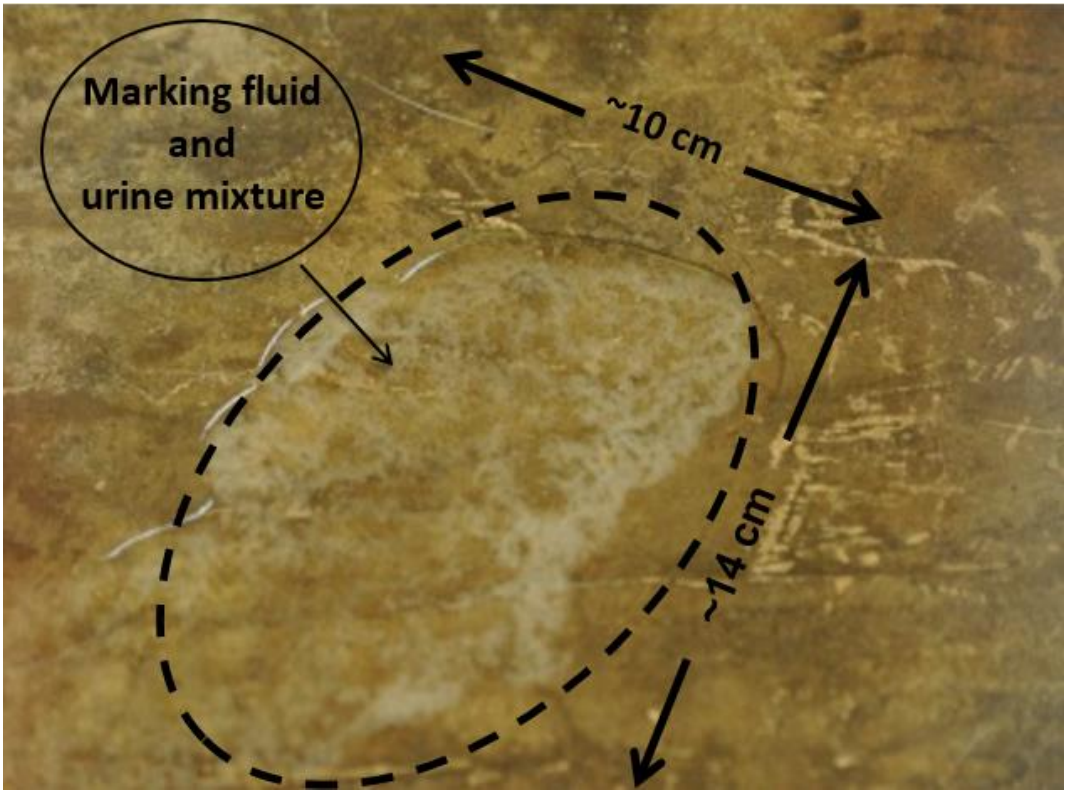

Supplementary Figure S1. Lion Marking Fluid. Marking fluid and urine mixture released unto the floor of the indoor enclosure by a male in a squatting downward position. The urine appeared to be yellow in color and the marking fluid had a whitish coloring.

Fiber Type	Size (diameter x length)	Target Analyte Description
**50/30 µm Divinylbenzene/Carboxen/ Polydimethylsiloxane	23 gauge x 2 cm	Broad range of analytes; Flavor compounds; Volatiles and Semi-volatiles; C3-C20 (MW 40-275)
50/30 µm Divinylbenzene/Carboxen/ Polydimethylsiloxane	24 gauge x 1 cm	Broad range of analytes; Flavor compounds; Volatiles and Semi-volatiles, C3-C20 (MW 40-275)
65 µm Polydimethylsiloxane/ Divinylbenzene	24 gauge x 1 cm	Volatiles; Amines; Nitro-aromatic compounds (MW 50-300)
75 µm Carboxen/ Polydimethylsiloxane	24 gauge x 1 cm	Volatile/ low molar mass analytes; Biogenic volatile organic compounds (MW 30-225)

**Fiber type selected for the rest of the study

Supplementary Table S4. SPME fiber type selection. Fiber types tested for extraction efficiency of characteristic *P. leo* scent marking odor compounds.
